# Supplementary material for: Exploring Novel Pyridine Carboxamide Derivatives as Urease Inhibitors: Synthesis, Molecular Docking, Kinetic Studies and ADME Profile
Source: Pharmaceuticals (Basel). 2022 Oct 19;15(10):1288. doi: 10.3390/ph15101288 (PMC9609714; doi:10.3390/ph15101288)
Supplement: Supplementary file 1 [file pharmaceuticals-15-01288-s001.zip › pharmaceuticals-1899372-supplementary.pdf]

Supplementary Materials:

Exploring Novel Pyridine Carboxamide Derivatives as Urease Inhibitors: Synthesis, Molecular Docking, Kinetic Studies and ADME Profile

RX- 1

IR

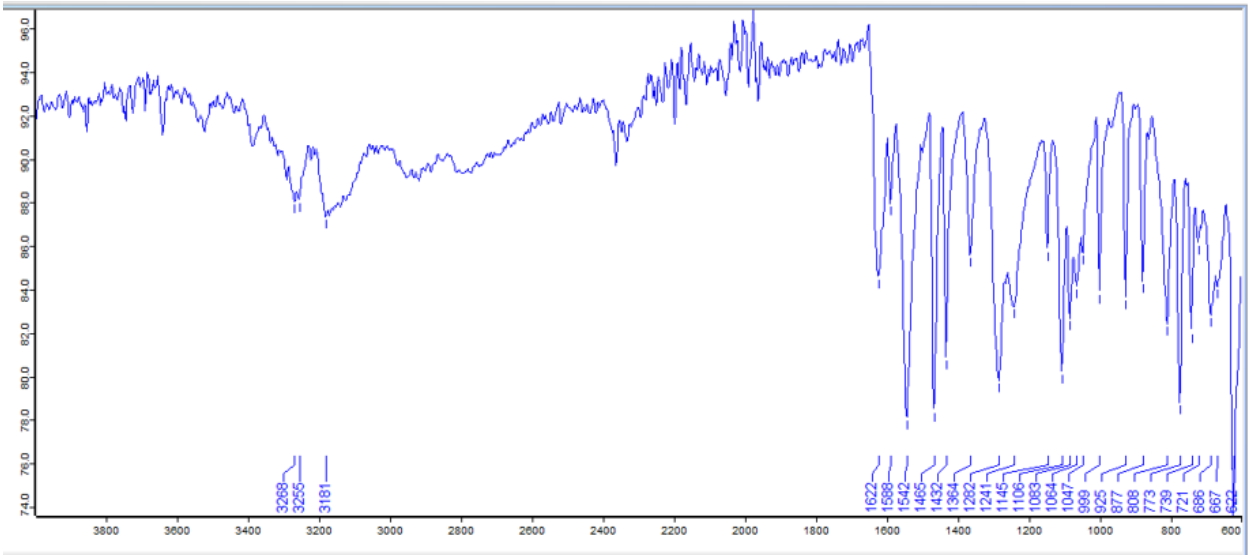

Mass

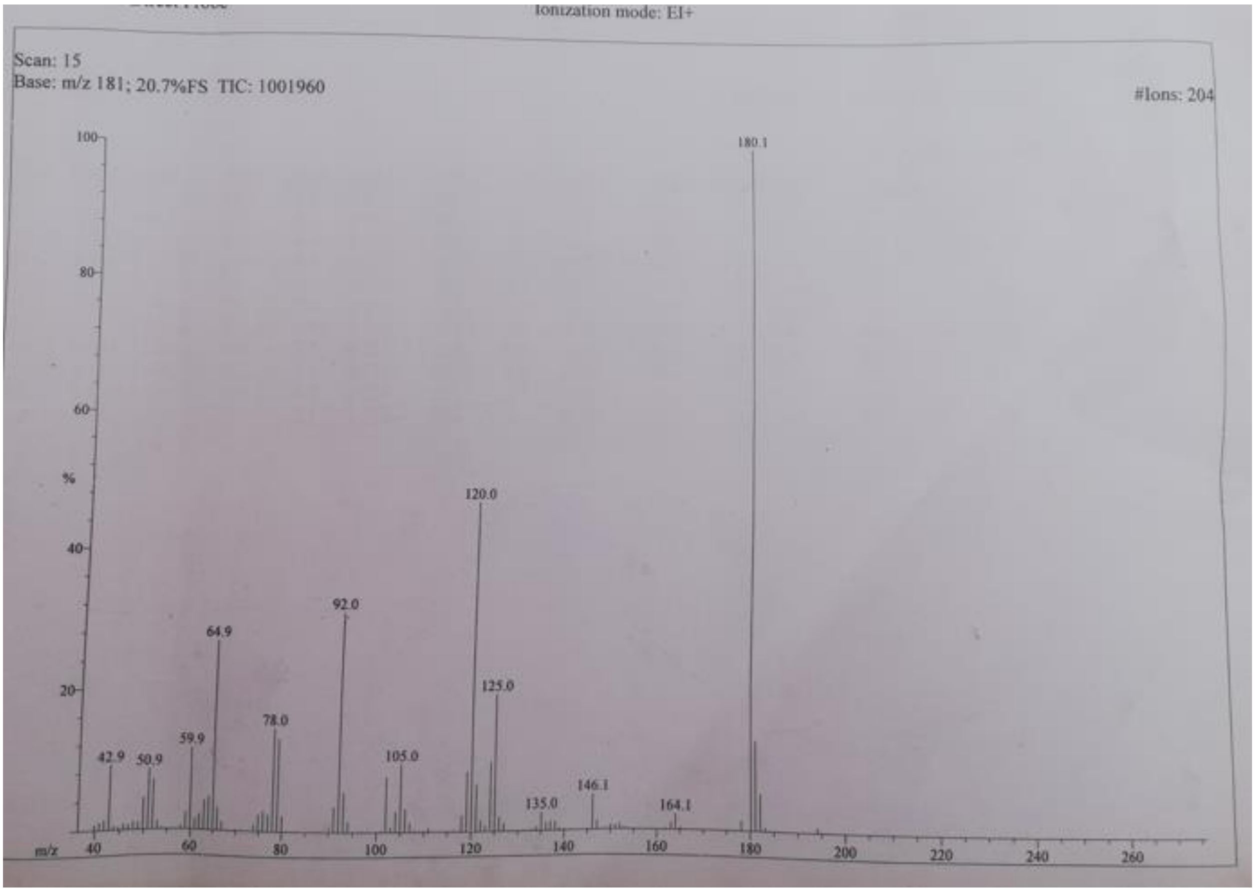
Ionization mode: EI+
Scan: 15
Base: m/z 181; 20.7%FS TIC: 1001960
#Ions: 204
180.1
120.0
92.0
64.9
125.0
78.0
59.9
42.9
50.9
105.0
146.1
135.0
164.1
m/z

1H NMR

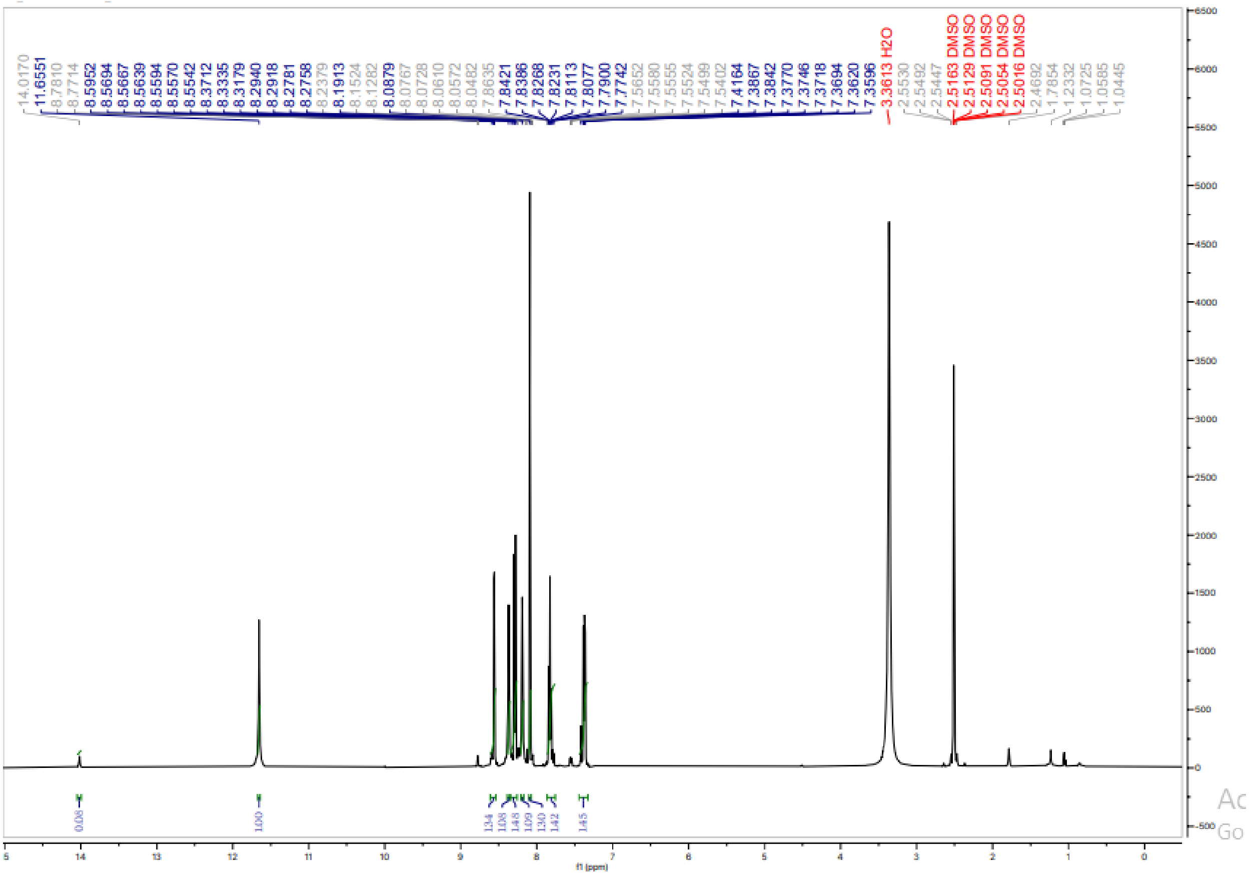
3.3613 H2O
2.5163 DMSO
2.5129 DMSO
2.5091 DMSO
2.5054 DMSO
2.5016 DMSO
f1 (ppm)

13C NMR

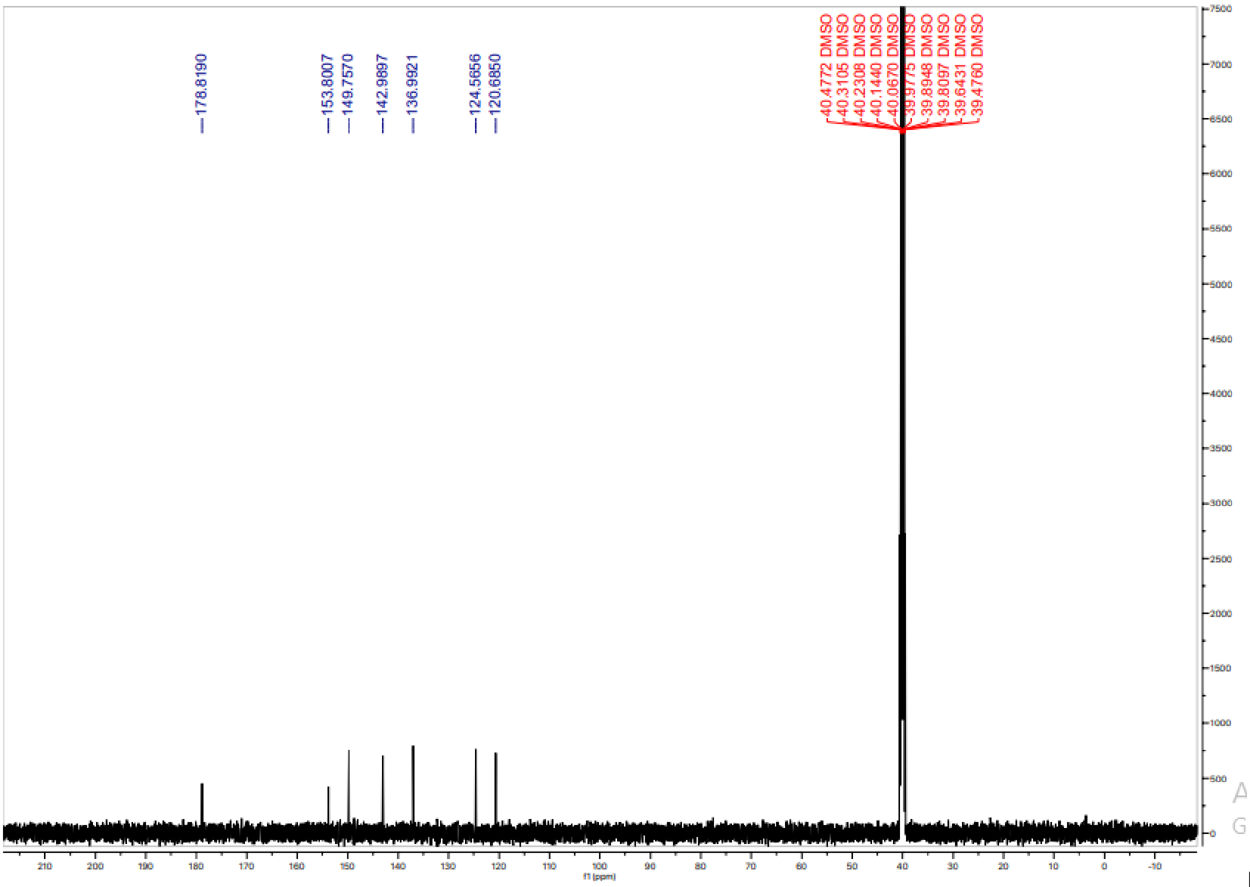

RX-2

IR

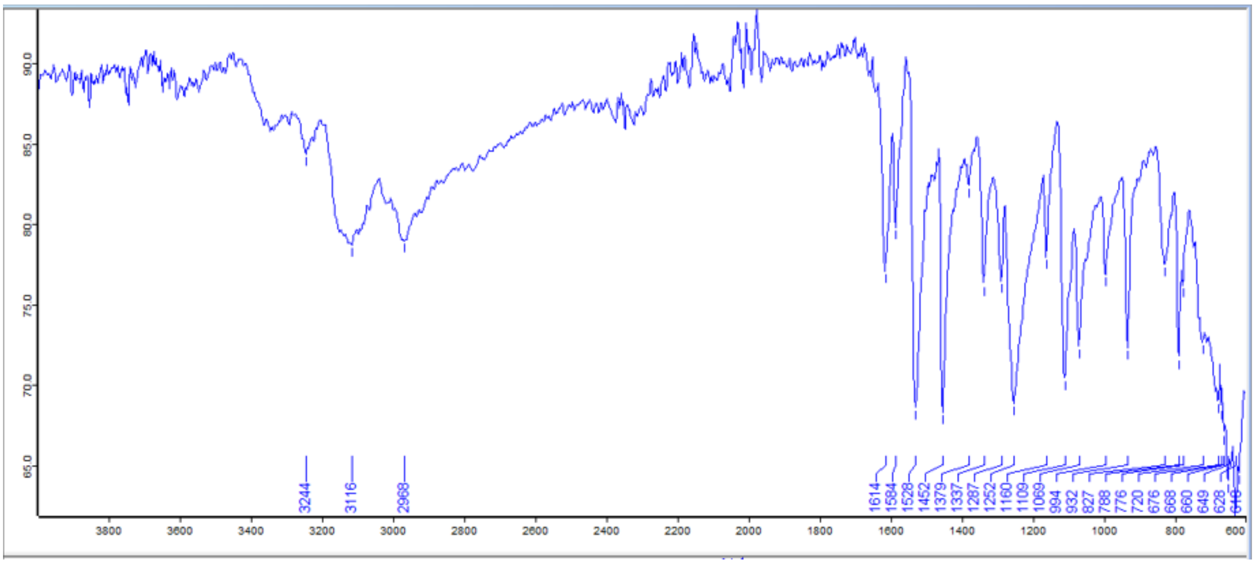

Mass

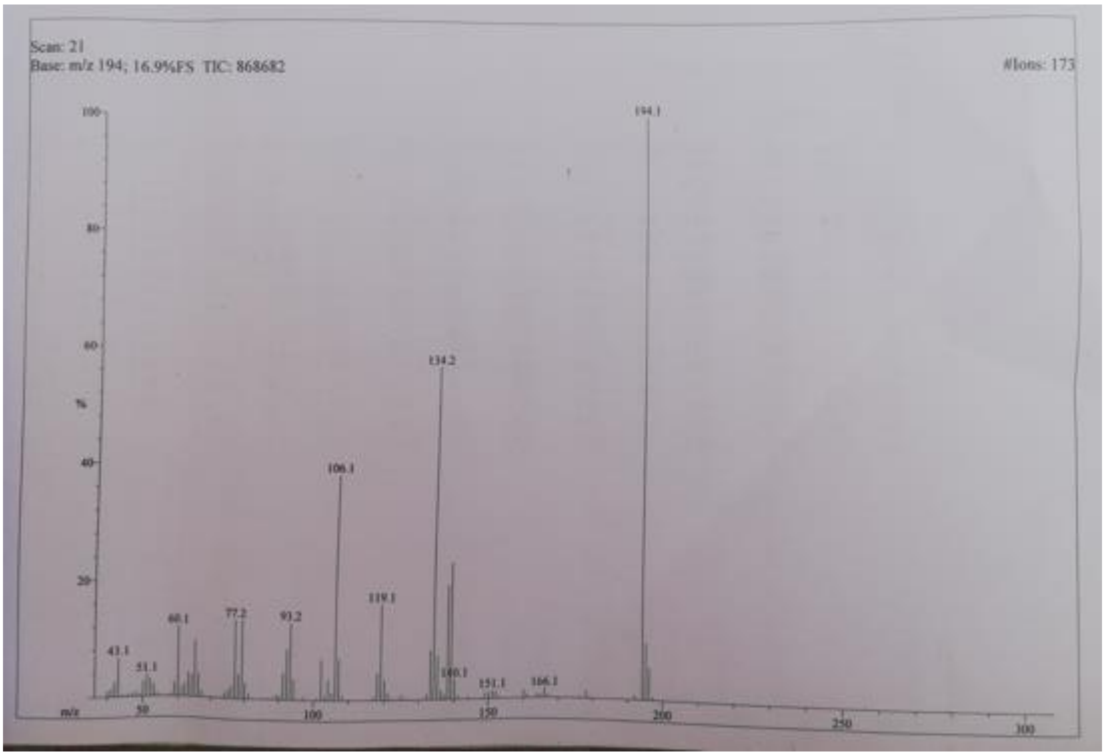
Scan: 21
Base: m/z 194; 16.9%FS TIC: 868682
#Ions: 173
194.1
134.2
106.1
119.1
60.1
77.2
93.2
43.1
51.1
140.1
151.1
166.1
m/z
%

NMR

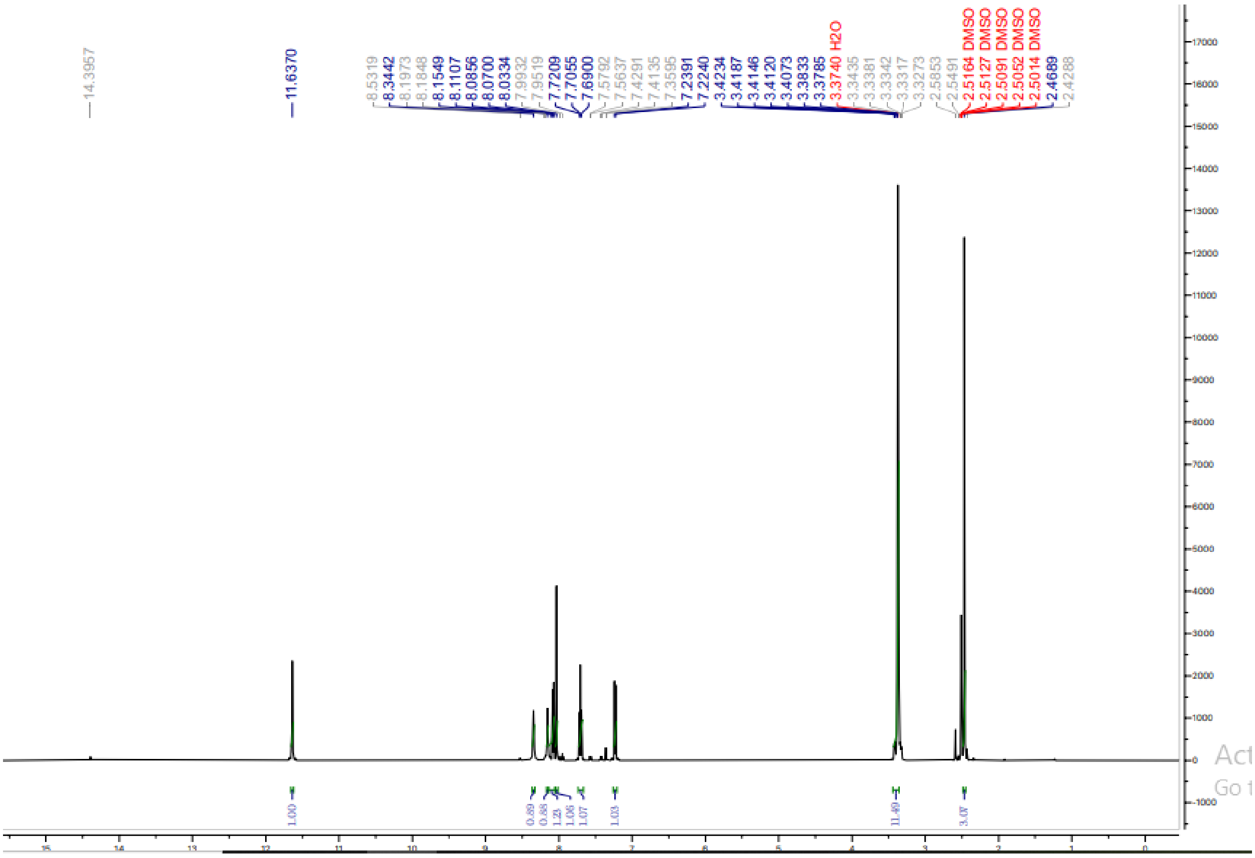
14.3957
11.6370
3.3740 H2O
2.5164 DMSO
2.5127 DMSO
2.5091 DMSO
2.5052 DMSO
2.5014 DMSO
1.00
11.49
3.07

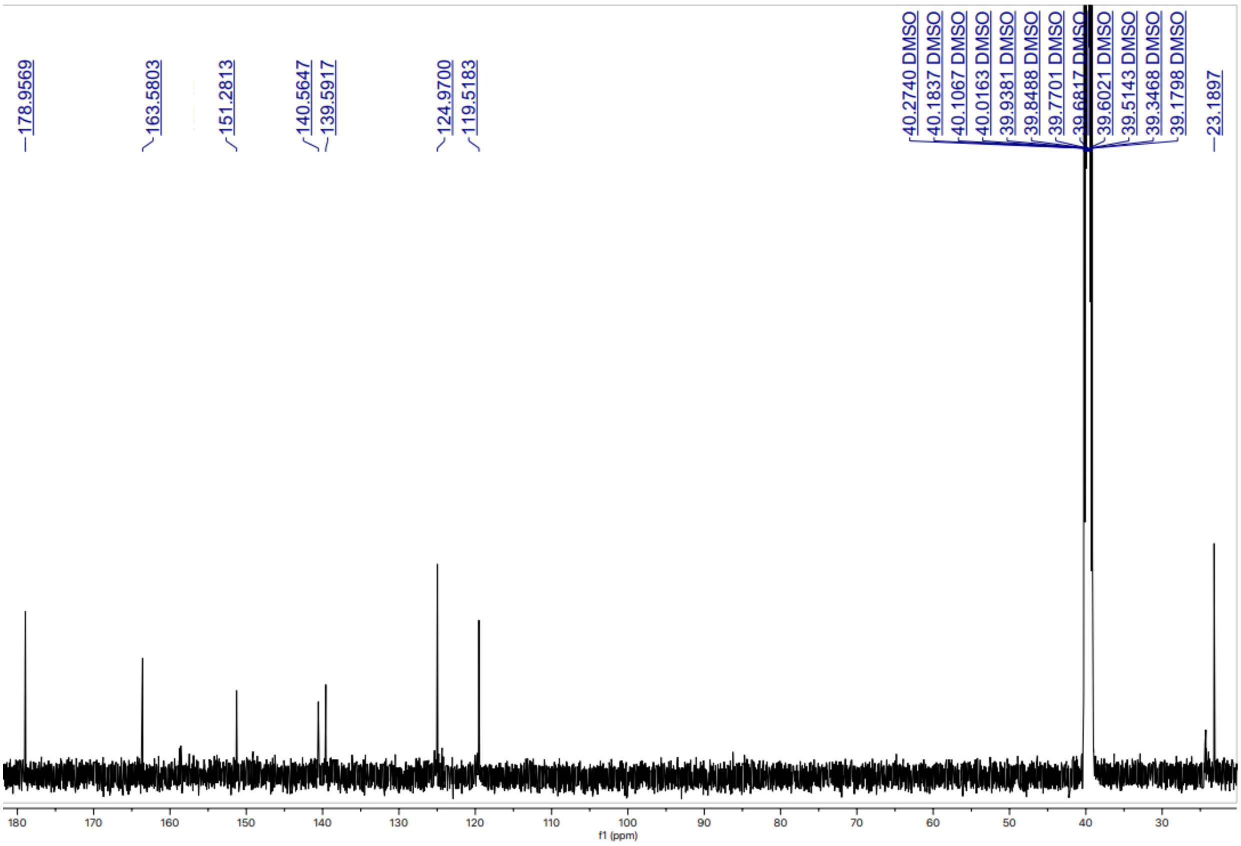
178.9569
163.5803
151.2813
140.5647
139.5917
124.9700
119.5183
40.2740 DMSO
40.1837 DMSO
40.1067 DMSO
40.0163 DMSO
39.9381 DMSO
39.8488 DMSO
39.7701 DMSO
39.6817 DMSO
39.6021 DMSO
39.5143 DMSO
39.3468 DMSO
39.1798 DMSO
23.1897
180
170
160
150
140
130
120
110
100
f1 (ppm)
90
80
70
60
50
40
30

R-3

IR

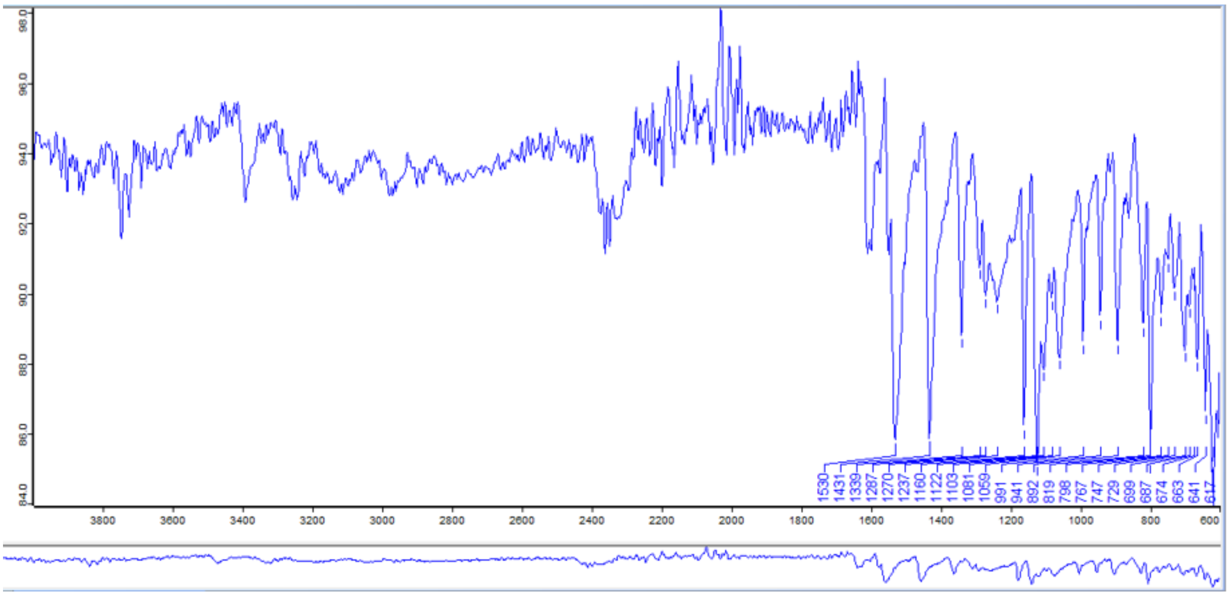
98.0
96.0
94.0
92.0
90.0
88.0
86.0
84.0
3800
3600
3400
3200
3000
2800
2600
2400
2200
2000
1800
1600
1400
1200
1000
800
600
1530
1431
1339
1287
1270
1237
1160
1122
1103
1081
1059
991
941
892
819
798
767
747
729
699
687
674
663
641
617

Mass

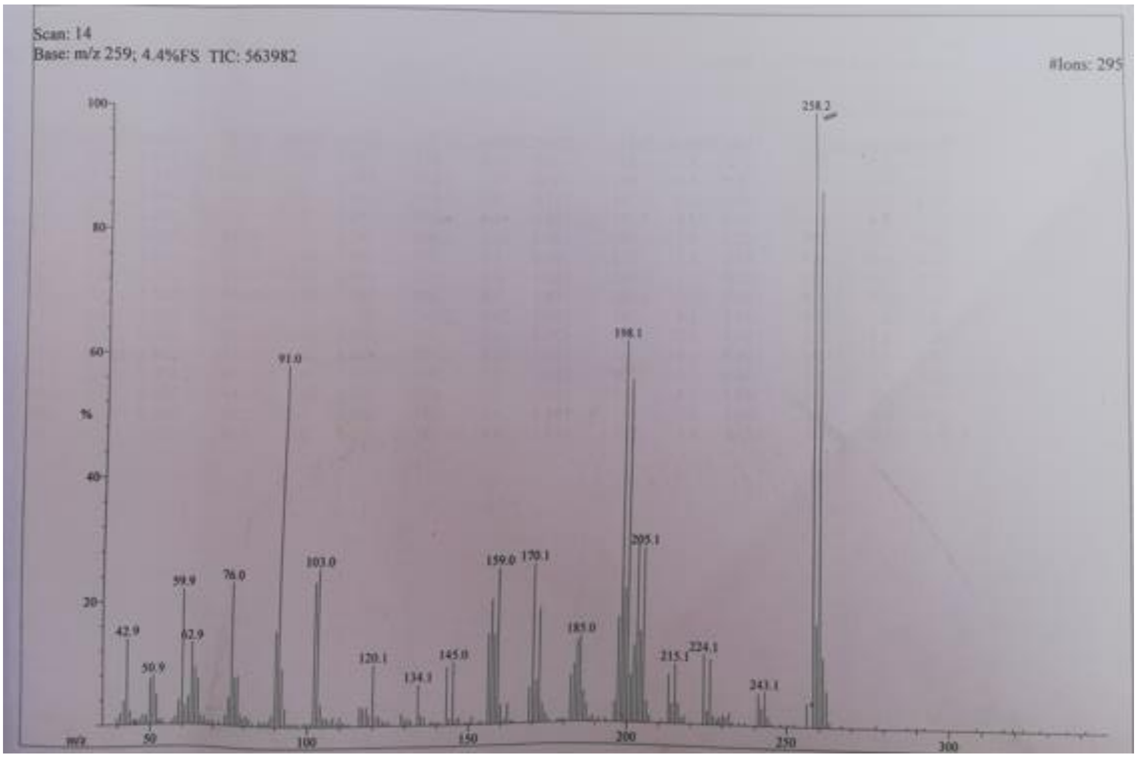
Scan: 14
Base: m/z 259; 4.4%FS TIC: 563982
#Ions: 295
258.2
198.1
91.0
205.1
103.0
159.0
170.1
76.0
59.9
185.0
62.9
42.9
224.1
215.1
120.1
145.0
50.9
134.1
243.1

NMR

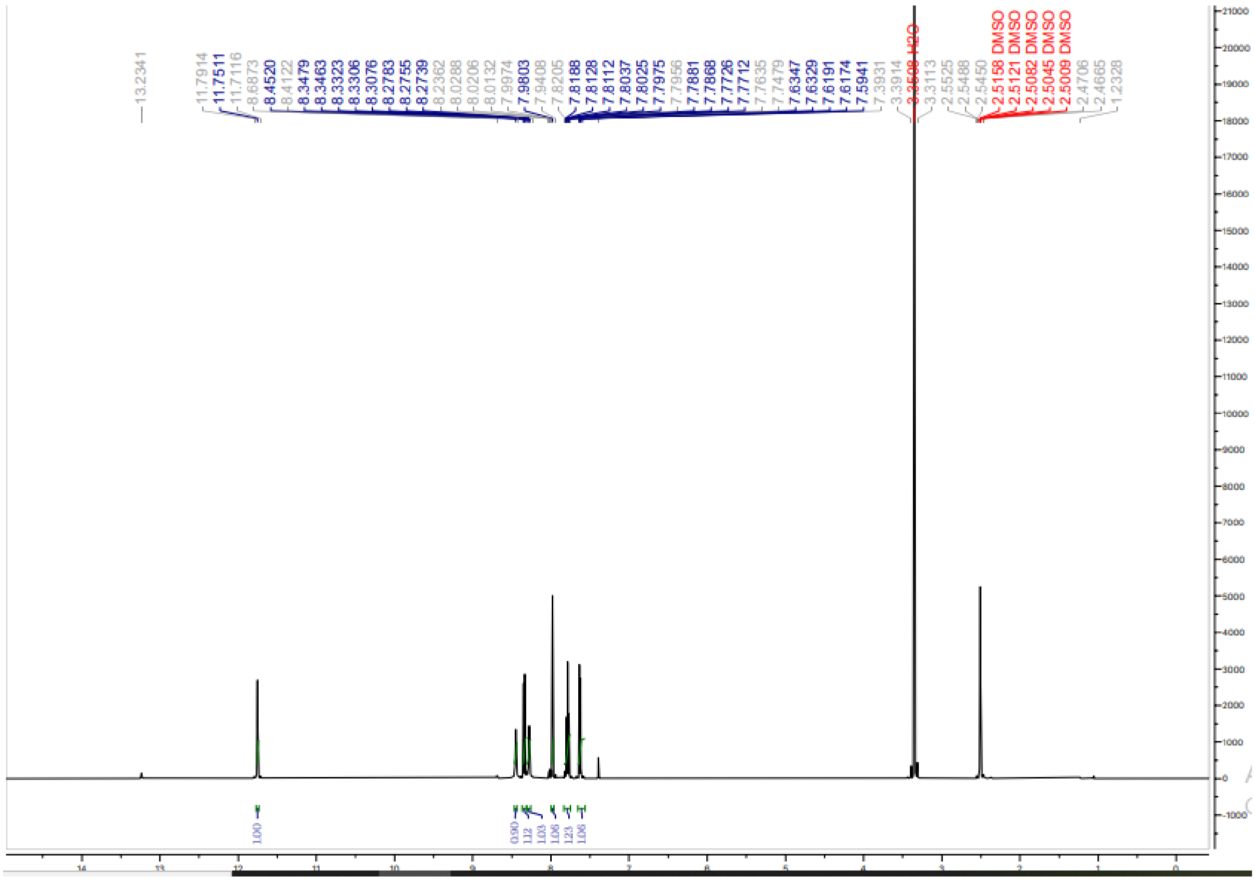

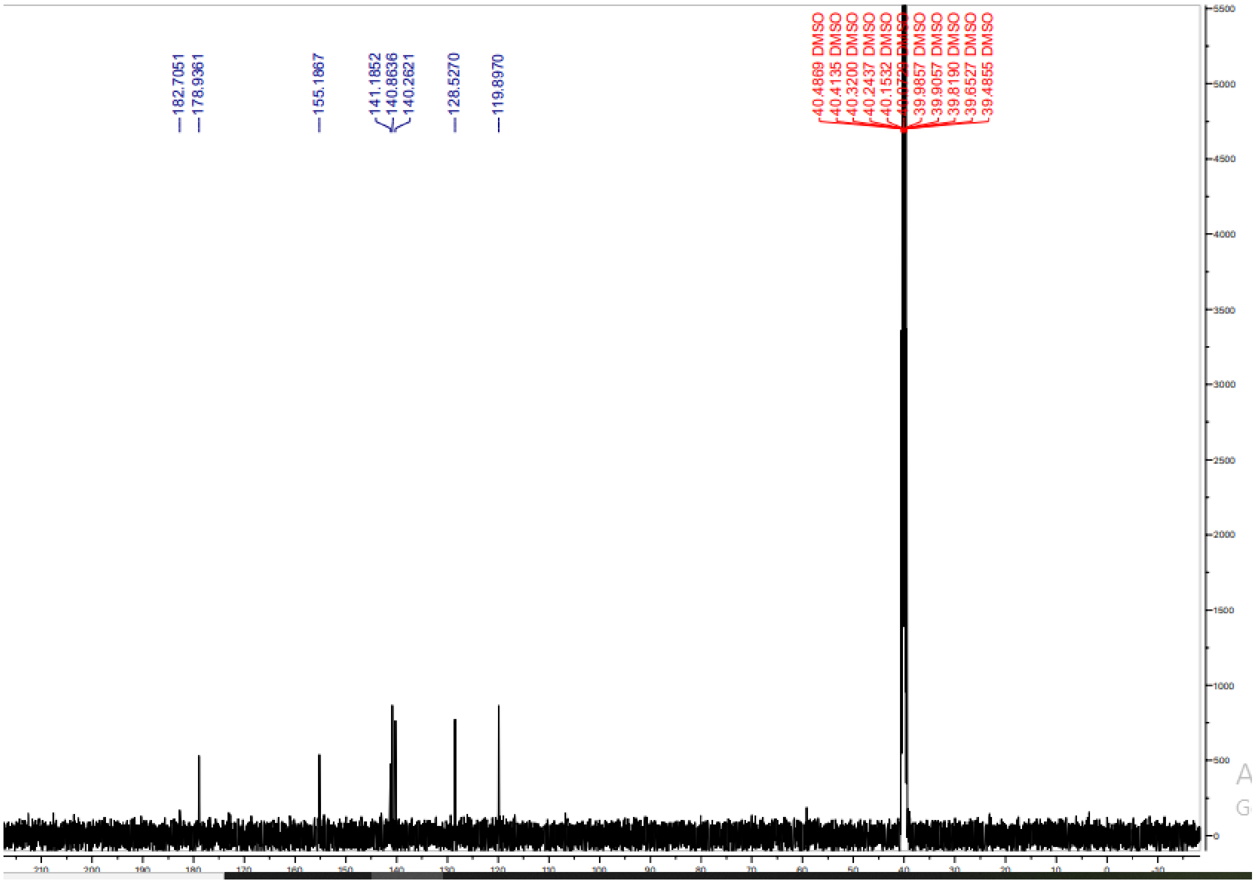
182.7051
178.9361
155.1867
141.1852
140.8636
140.2621
128.5270
119.8970
40.4869 DMSO
40.4135 DMSO
40.3200 DMSO
40.2437 DMSO
40.1532 DMSO
39.9857 DMSO
39.9057 DMSO
39.8190 DMSO
39.6527 DMSO
39.4855 DMSO

Rx-4

IR

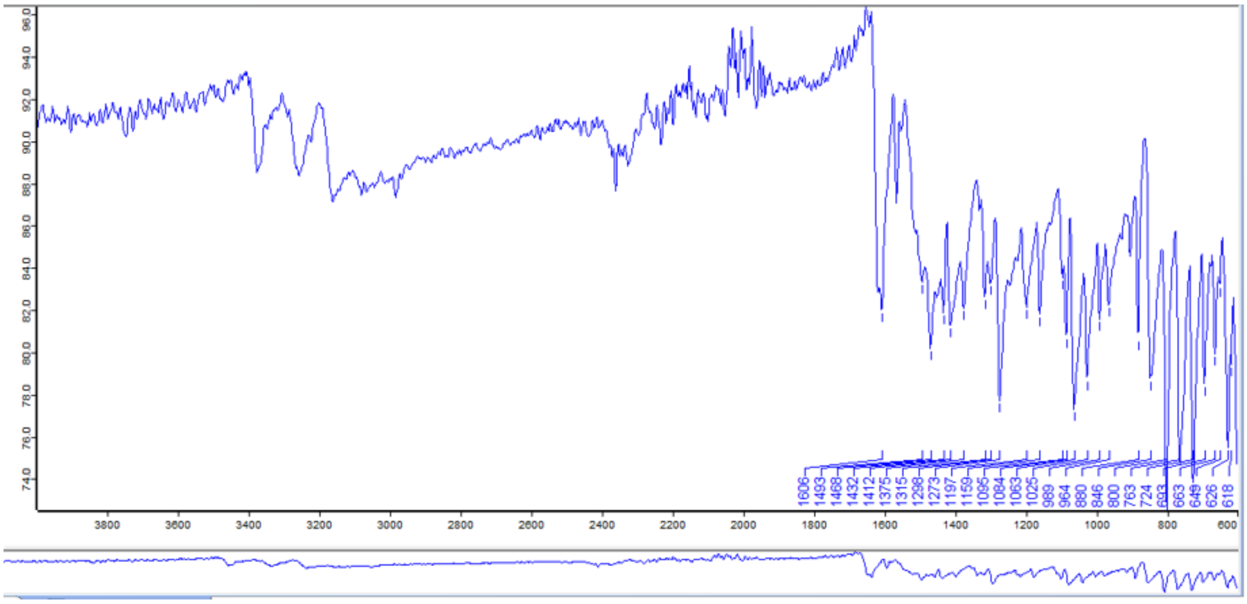
1606
1493
1468
1432
1412
1375
1315
1298
1273
1197
1159
1095
1084
1063
1025
989
964
880
846
800
763
724
693
663
649
626
618

Mass

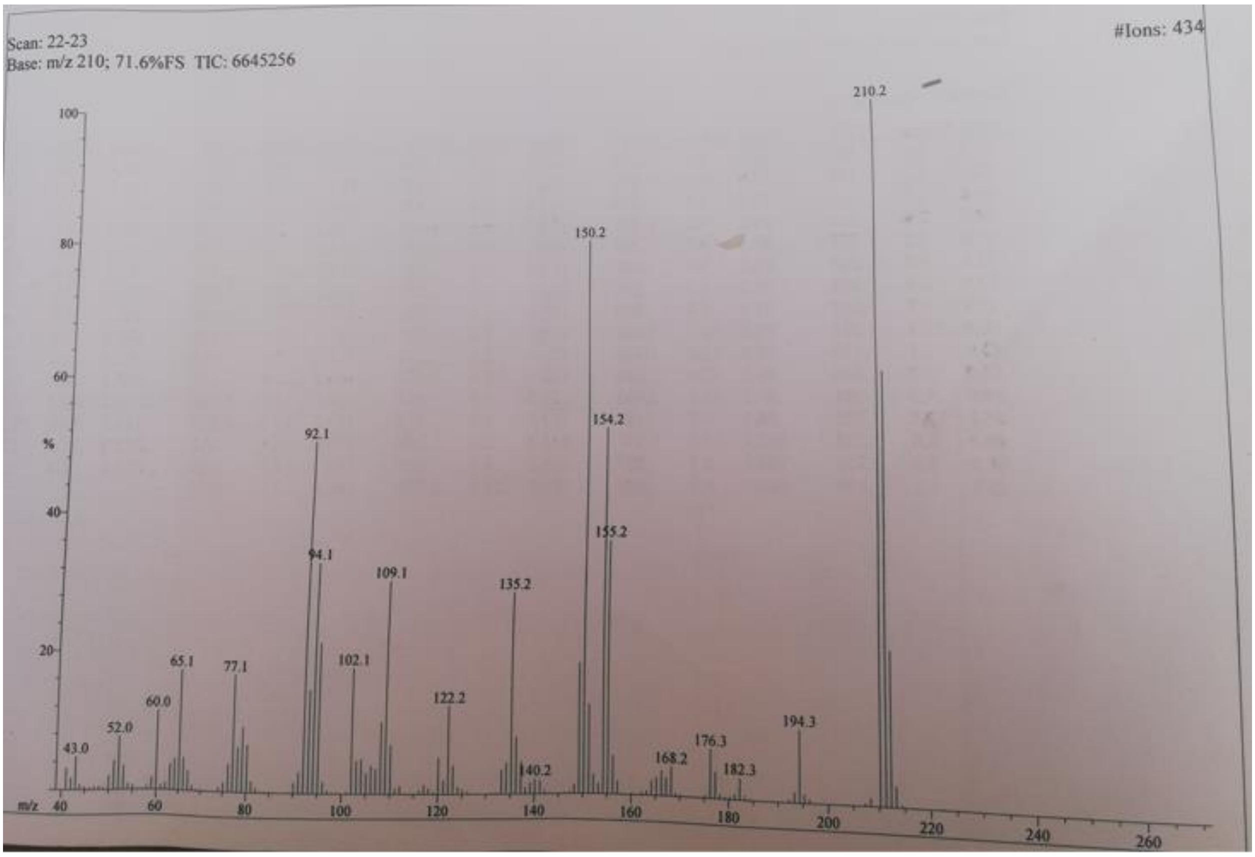
Scan: 22-23
Base: m/z 210; 71.6%FS TIC: 6645256
#Ions: 434
210.2
150.2
154.2
155.2
92.1
94.1
109.1
135.2
102.1
77.1
65.1
60.0
52.0
43.0
122.2
140.2
168.2
176.3
182.3
194.3
%
m/z

NMR

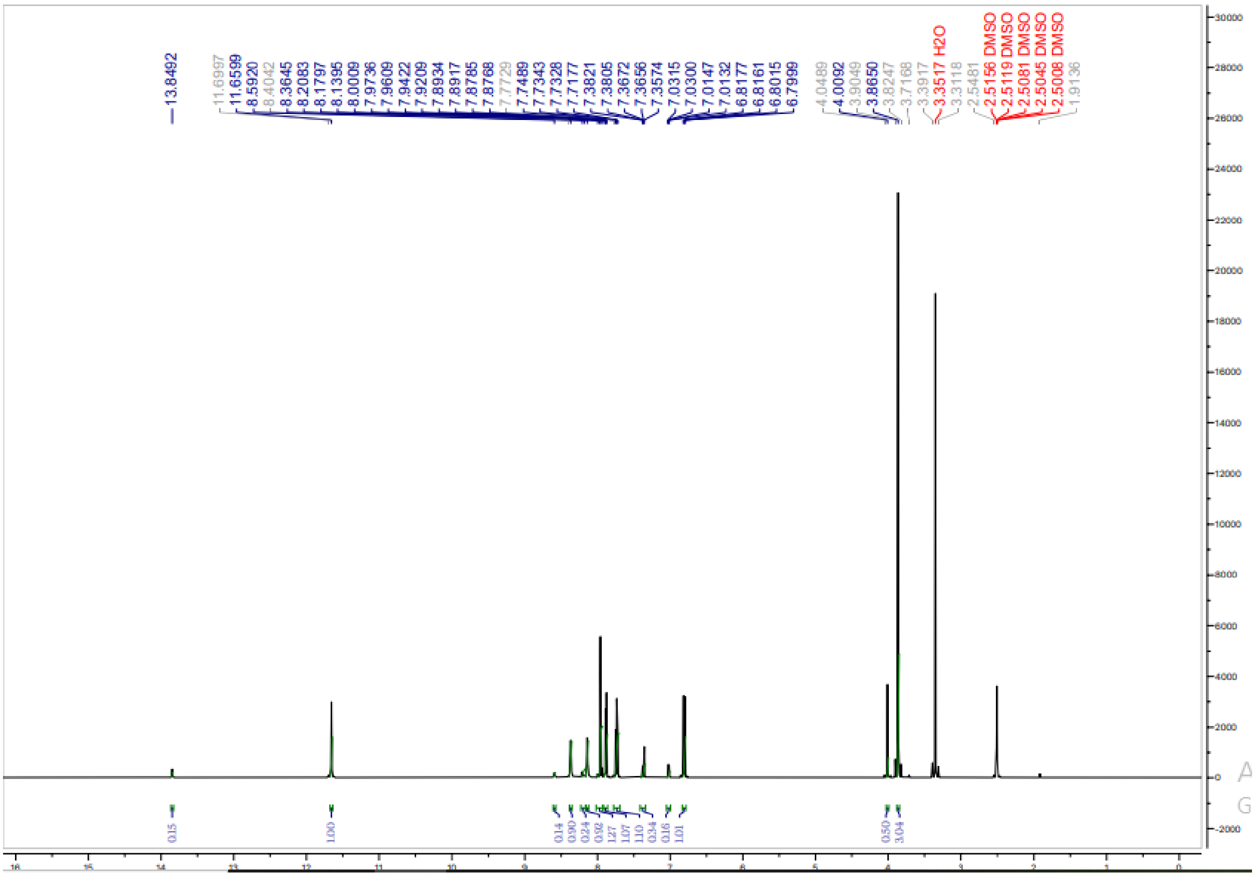
13.8492
11.6599
3.3517 H2O
2.5156 DMSO
2.5119 DMSO
2.5081 DMSO
2.5045 DMSO
2.5008 DMSO

Rx-5

IR

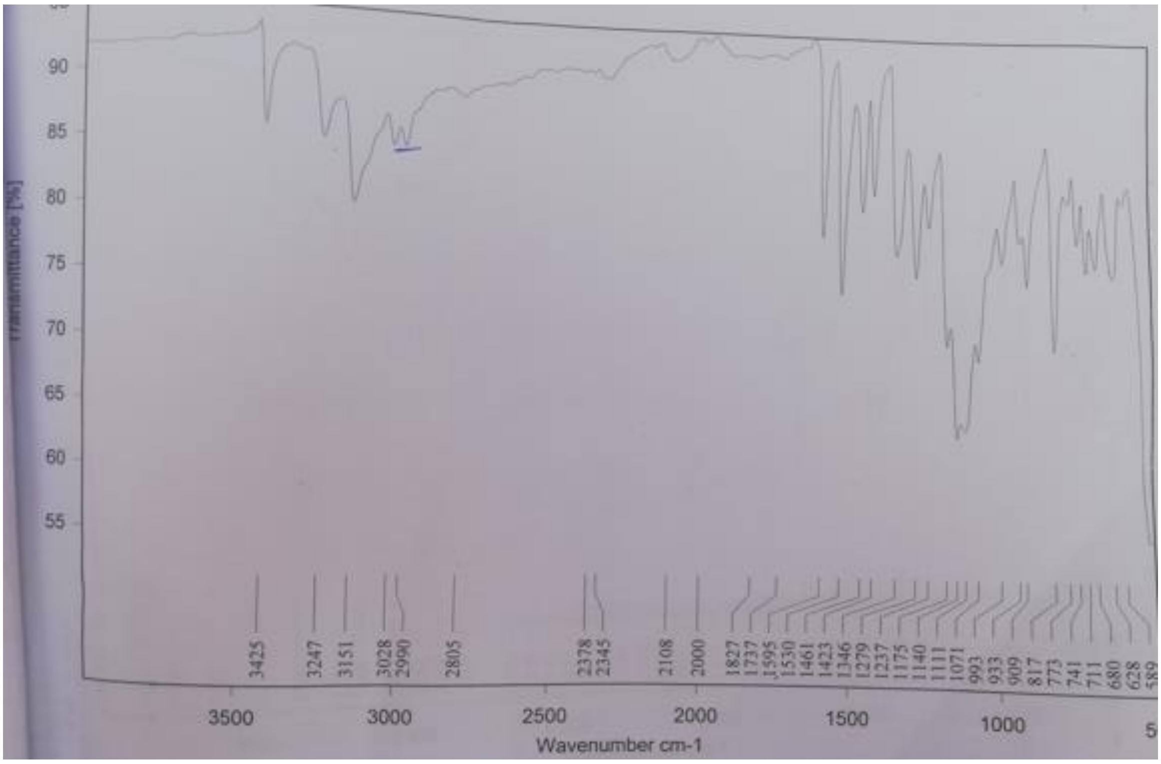

Transmittance [%]
90
85
80
75
70
65
60
55
3425
3247
3151
3028
2990
2805
2378
2345
2108
2000
1827
1737
1595
1530
1461
1423
1346
1279
1237
1175
1140
1111
1071
993
933
909
817
773
741
711
680
628
589
3500
3000
2500
2000
1500
1000
Wavenumber cm-1

Mass

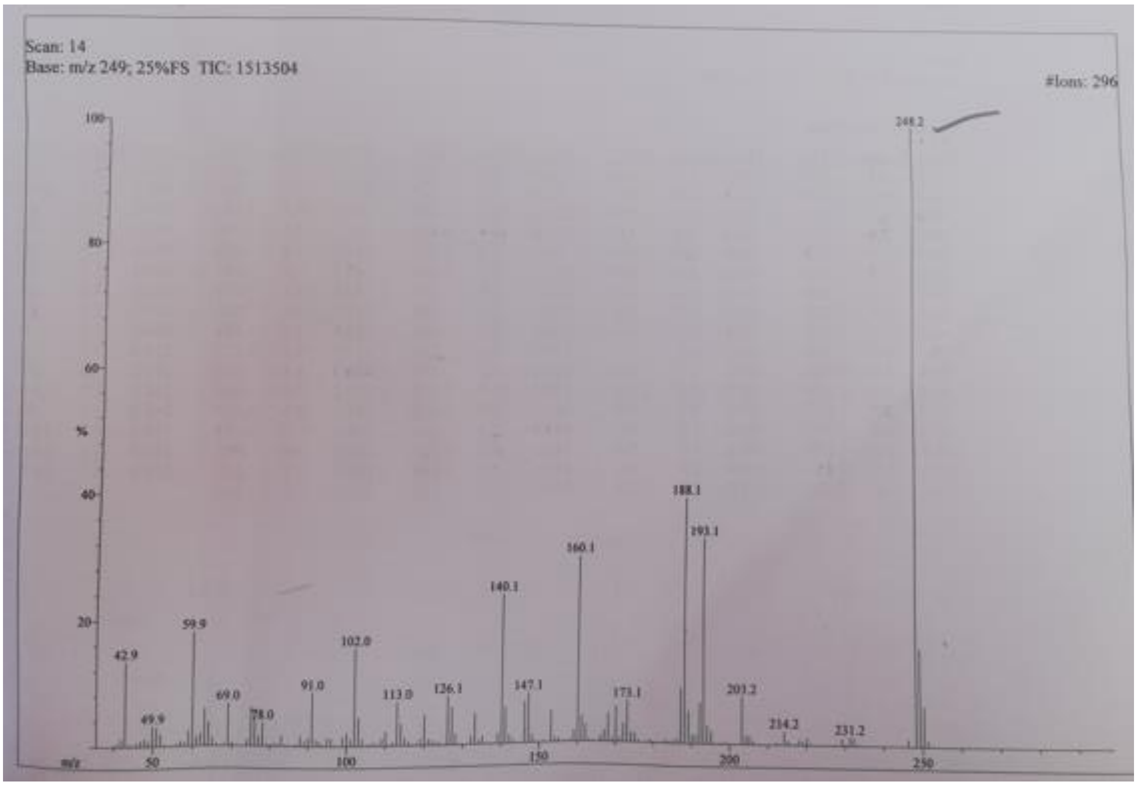

Scan: 14
Base: m/z 249; 25%FS TIC: 1513504
#Ions: 296
%
m/z
42.9
49.9
59.9
69.0
78.0
91.0
102.0
113.0
126.1
140.1
147.1
160.1
173.1
188.1
193.1
203.2
214.2
231.2
248.2
50
100
150
200
250

NMR

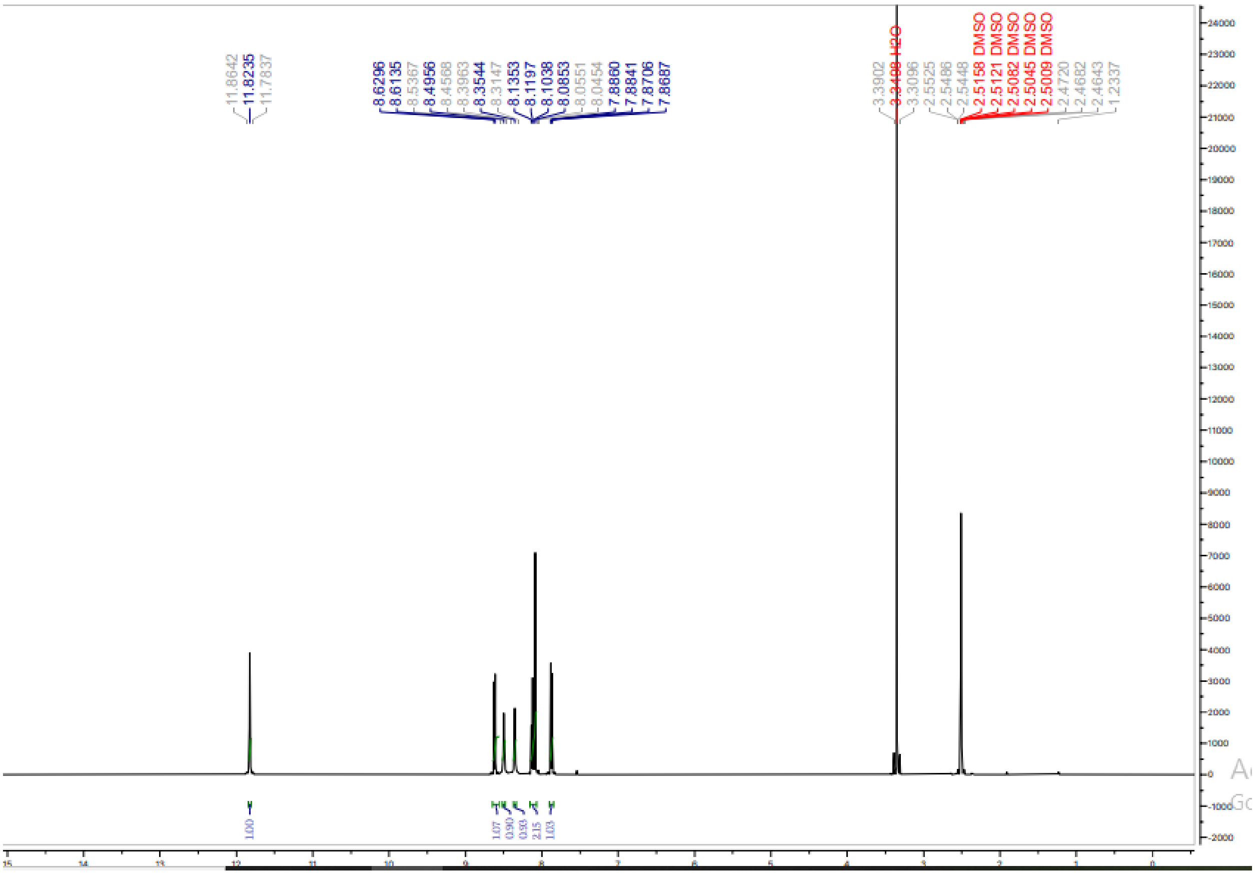

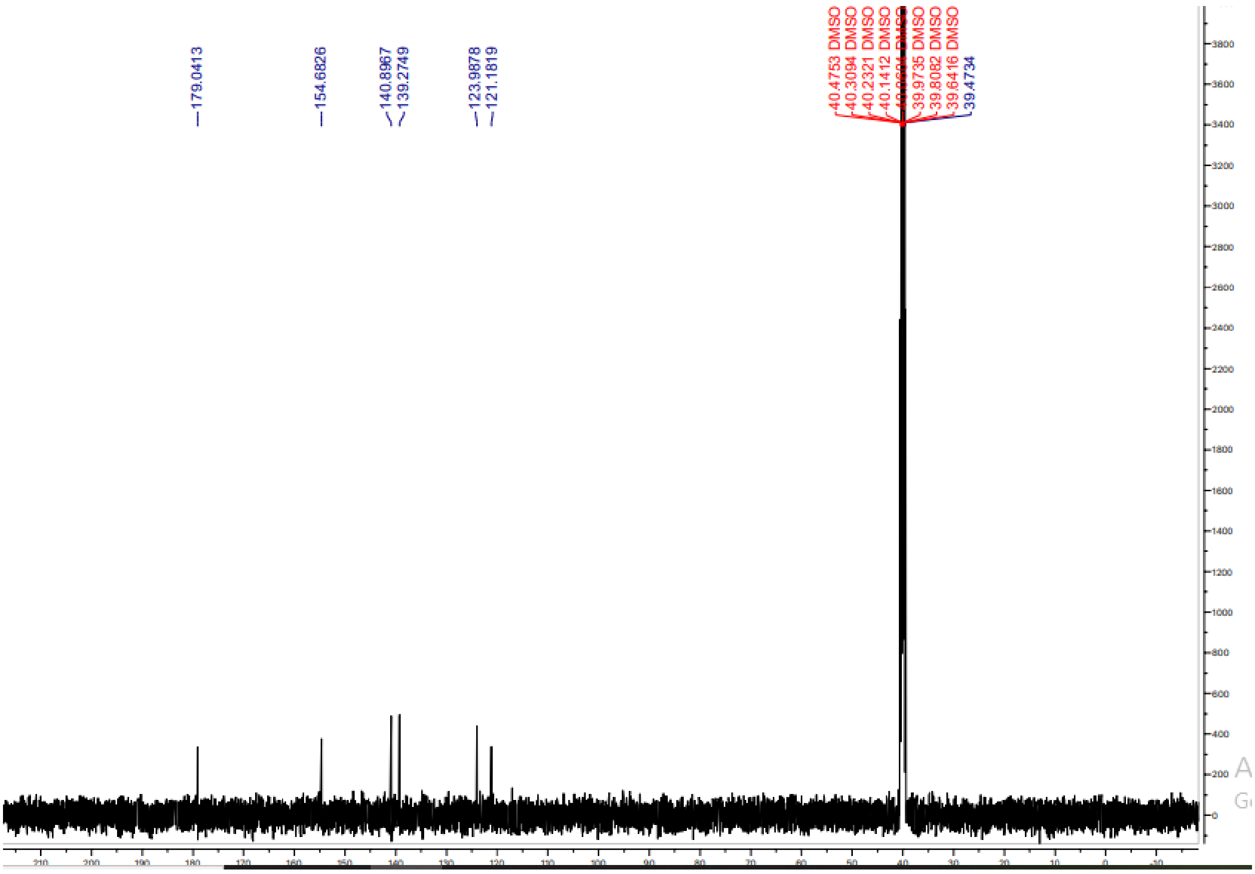

Rx-6

IR

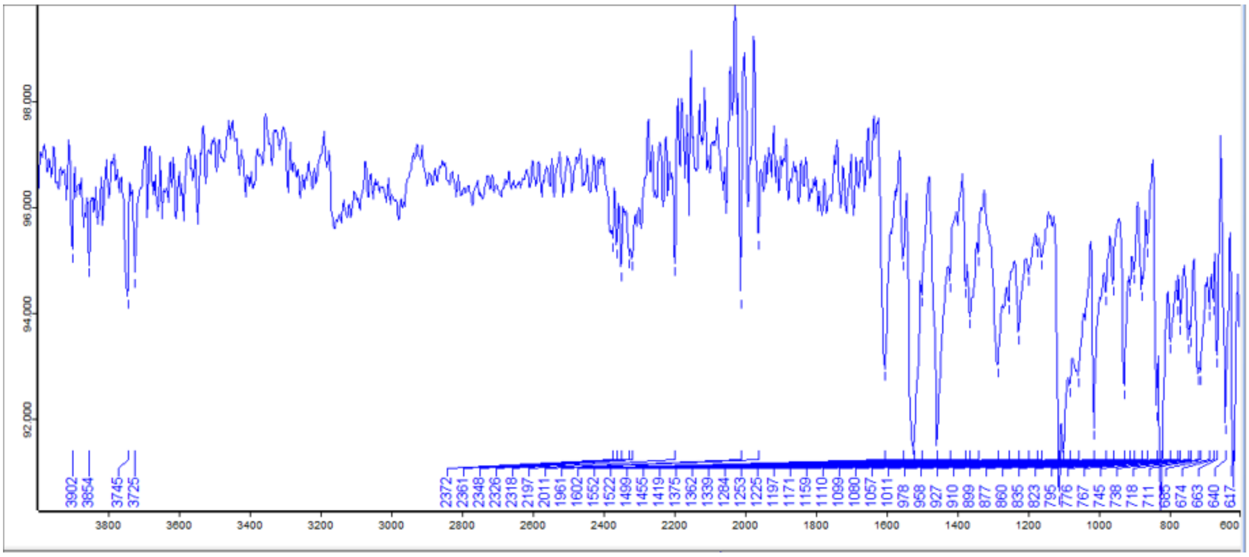

Mass

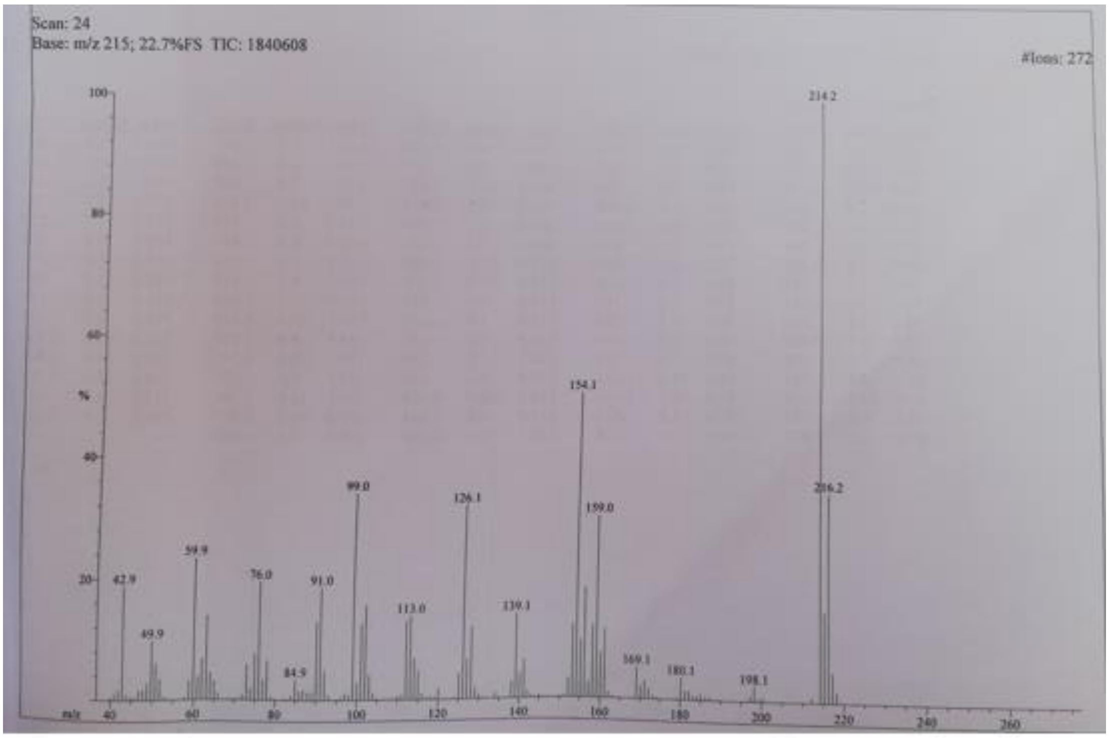

NMR

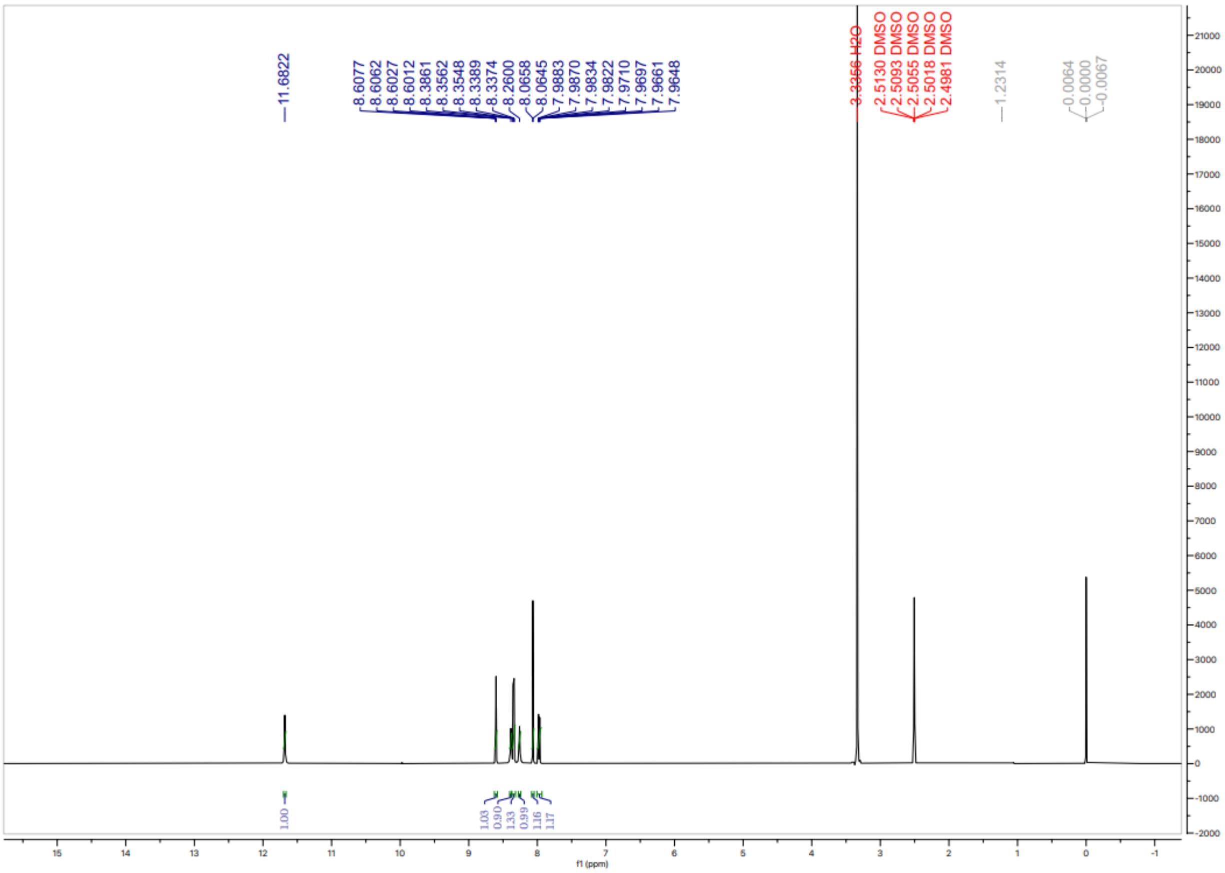
11.6822
8.6077
8.6062
8.6027
8.6012
8.3861
8.3562
8.3548
8.3389
8.3374
8.2600
8.0658
8.0645
7.9883
7.9870
7.9834
7.9822
7.9710
7.9697
7.9661
7.9648
3.3356 H2O
2.5130 DMSO
2.5093 DMSO
2.5055 DMSO
2.5018 DMSO
2.4981 DMSO
1.2314
0.0064
0.0000
-0.0067
f1 (ppm)

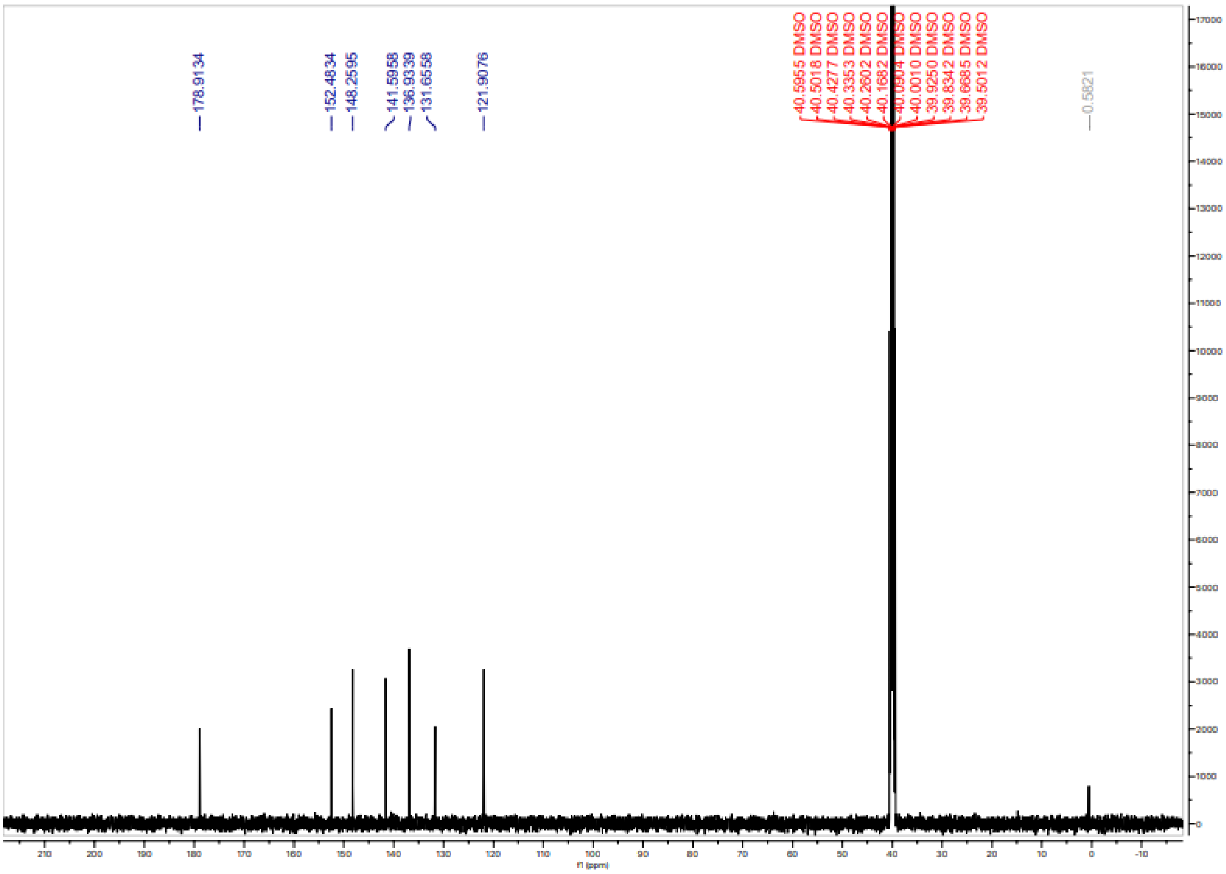
178.9134
152.4834
148.2595
141.5958
136.9339
131.6558
121.9076
40.5955 DMSO
40.5018 DMSO
40.4277 DMSO
40.3353 DMSO
40.2602 DMSO
40.1682 DMSO
40.0904 DMSO
40.0010 DMSO
39.9250 DMSO
39.8342 DMSO
39.6685 DMSO
39.5012 DMSO
-0.5821
f1 (ppm)

Rx-7

IR

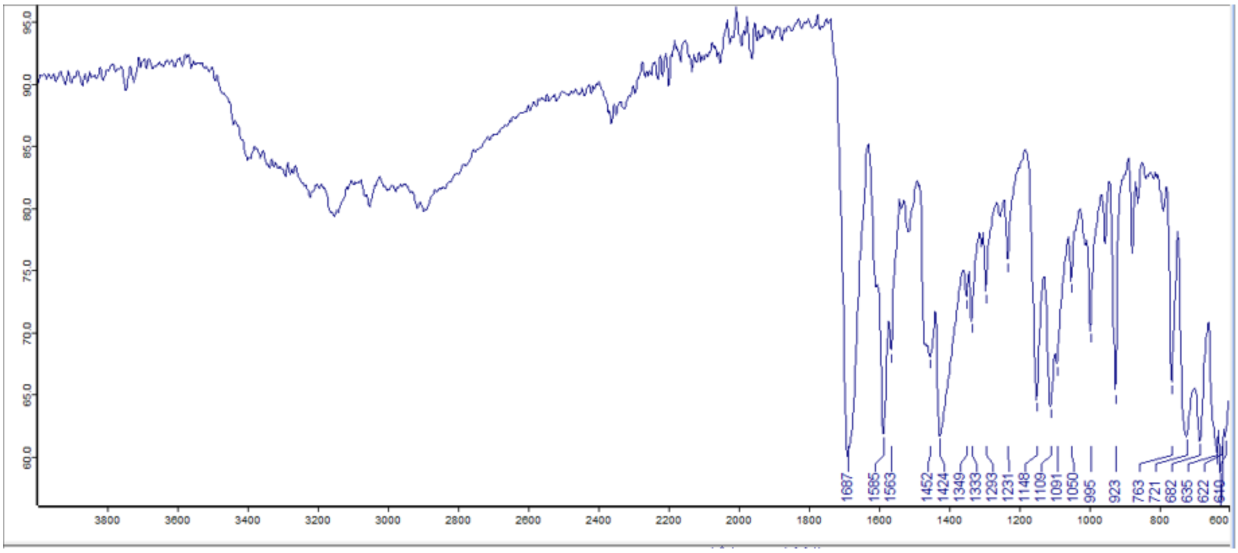

Mass

NMR

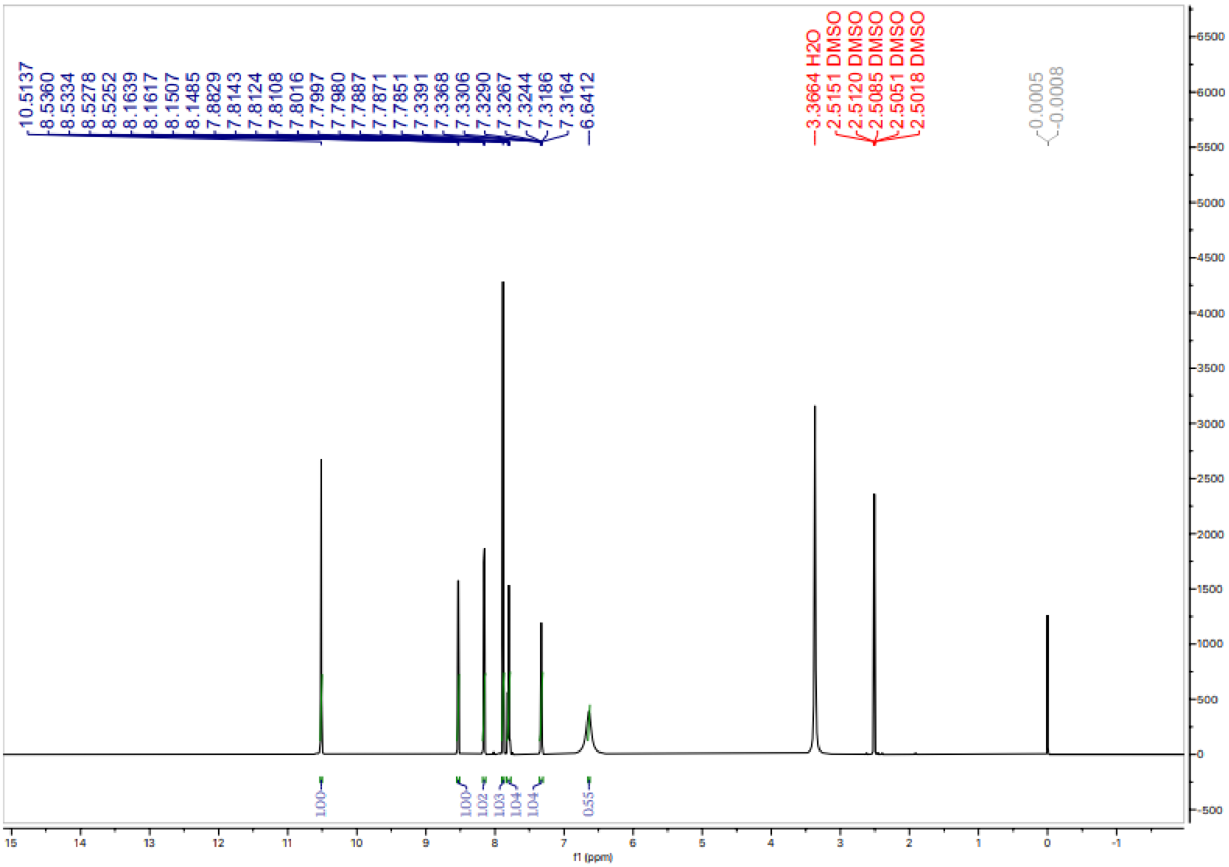

Rx-8

IR

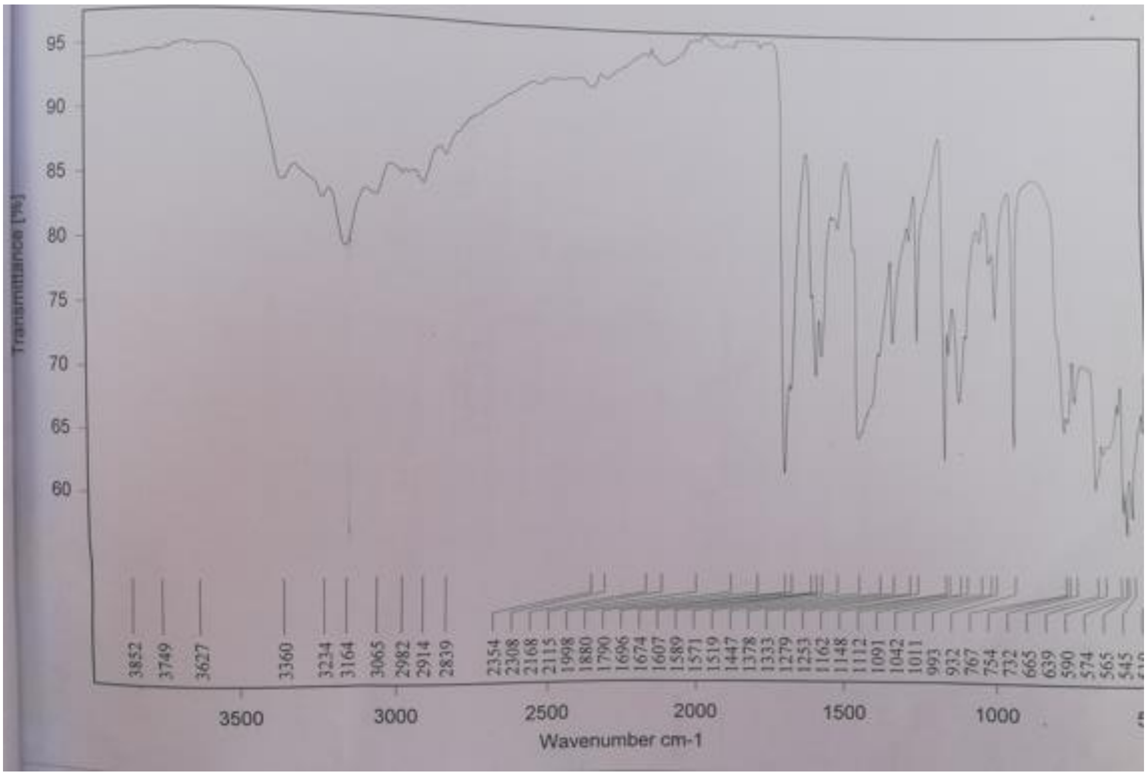

Mass

NMR

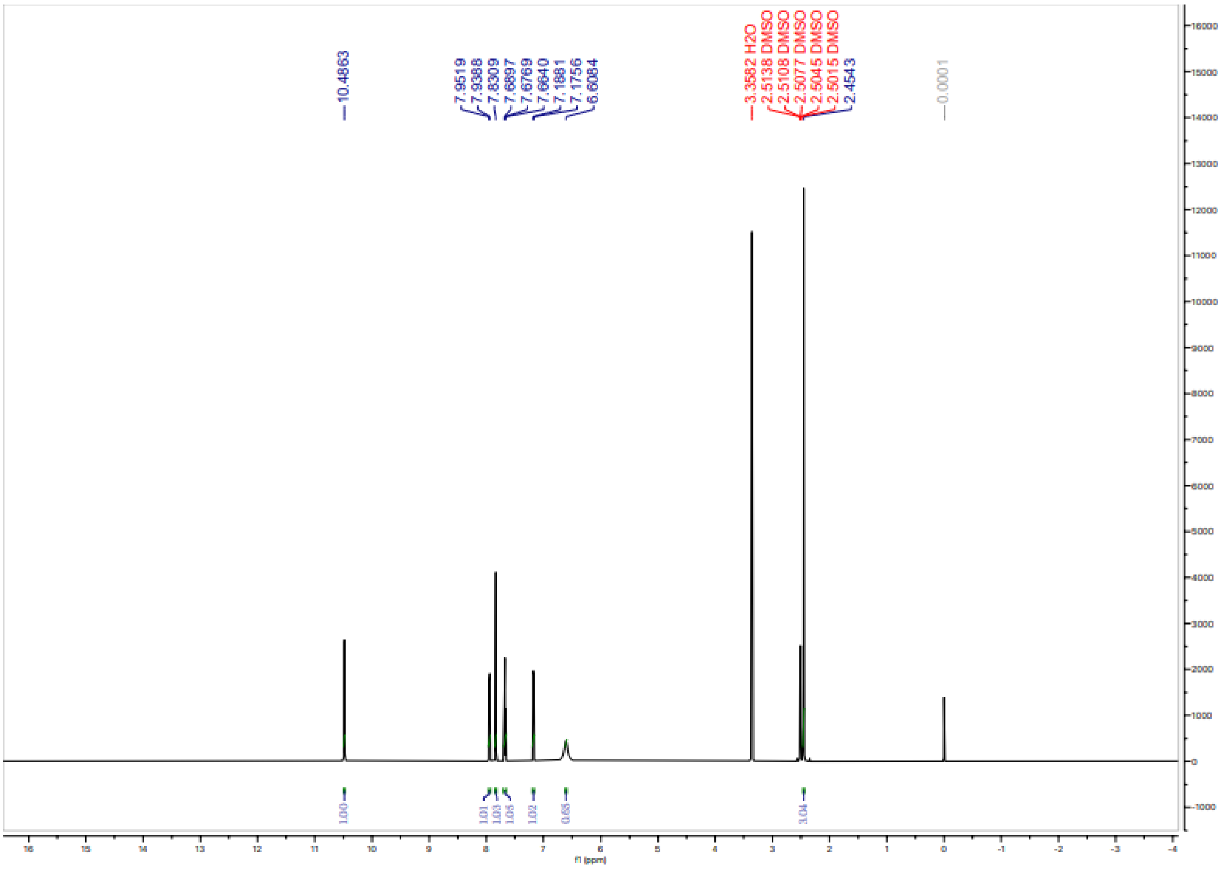

Rx-9

IR

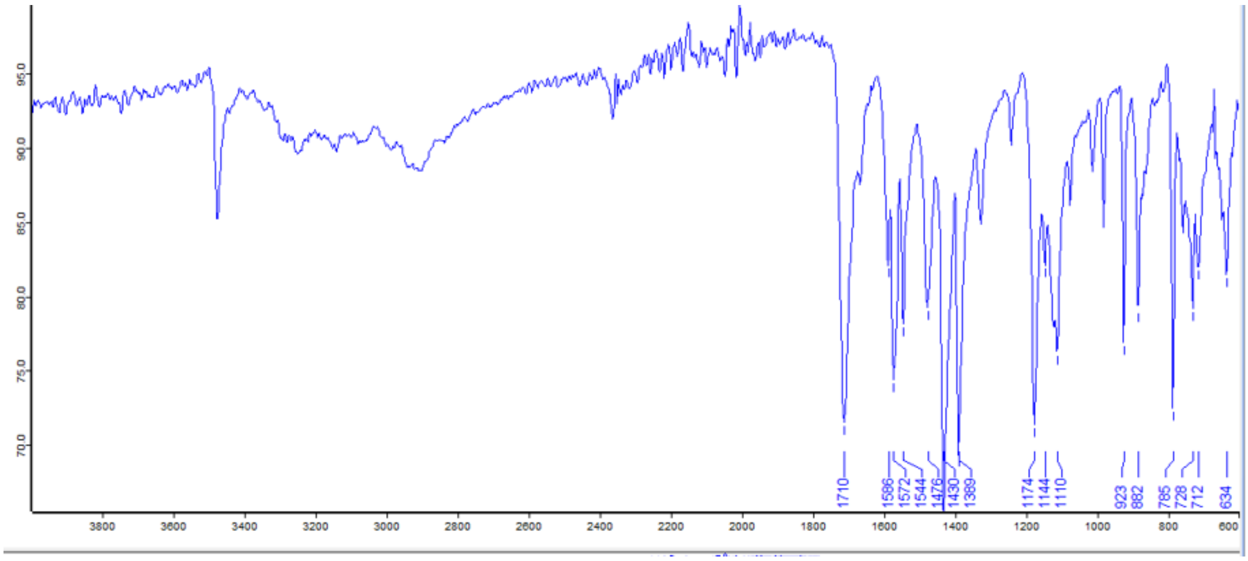
95.0
90.0
85.0
80.0
75.0
70.0
1710
1586
1572
1544
1476
1430
1389
1174
1144
1110
923
882
785
728
712
634
3800
3600
3400
3200
3000
2800
2600
2400
2200
2000
1800
1600
1400
1200
1000
800
600

Mass

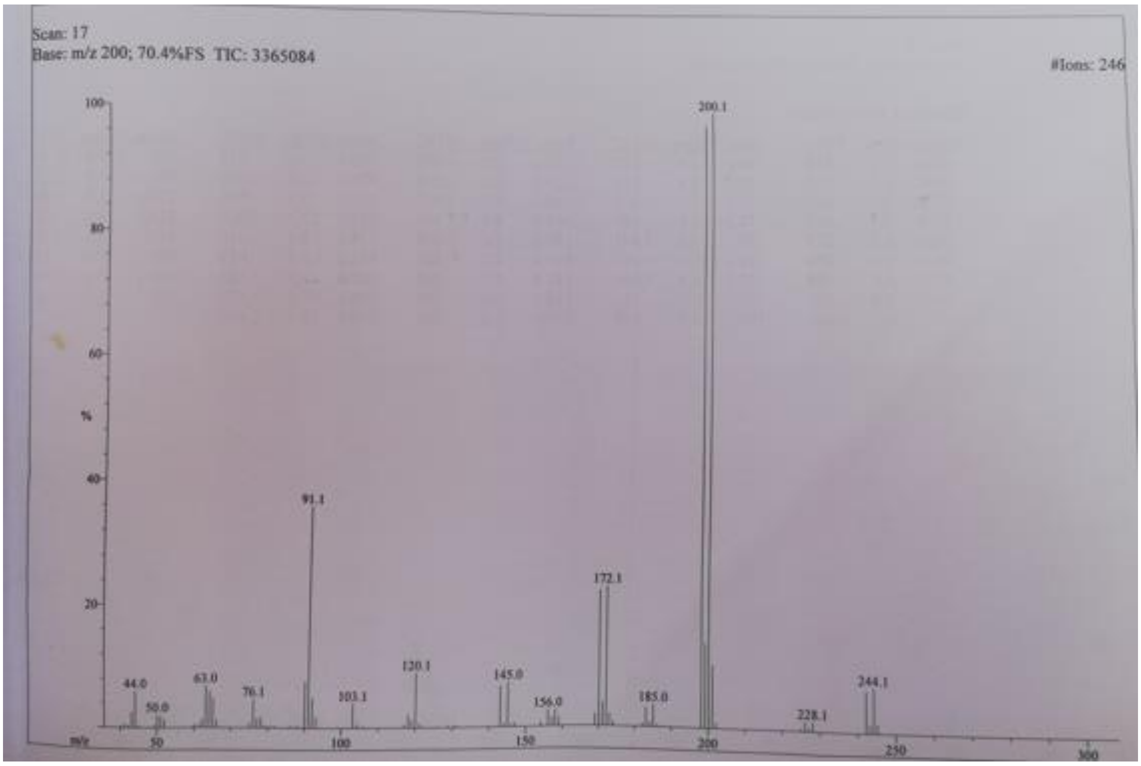
Scan: 17
Base: m/z 200; 70.4%FS TIC: 3365084
#Ions: 246
100
80
60
%
40
20
200.1
91.1
172.1
120.1
145.0
44.0
50.0
63.0
76.1
103.1
156.0
185.0
228.1
244.1
m/z
50
100
150
200
250
300

NMR

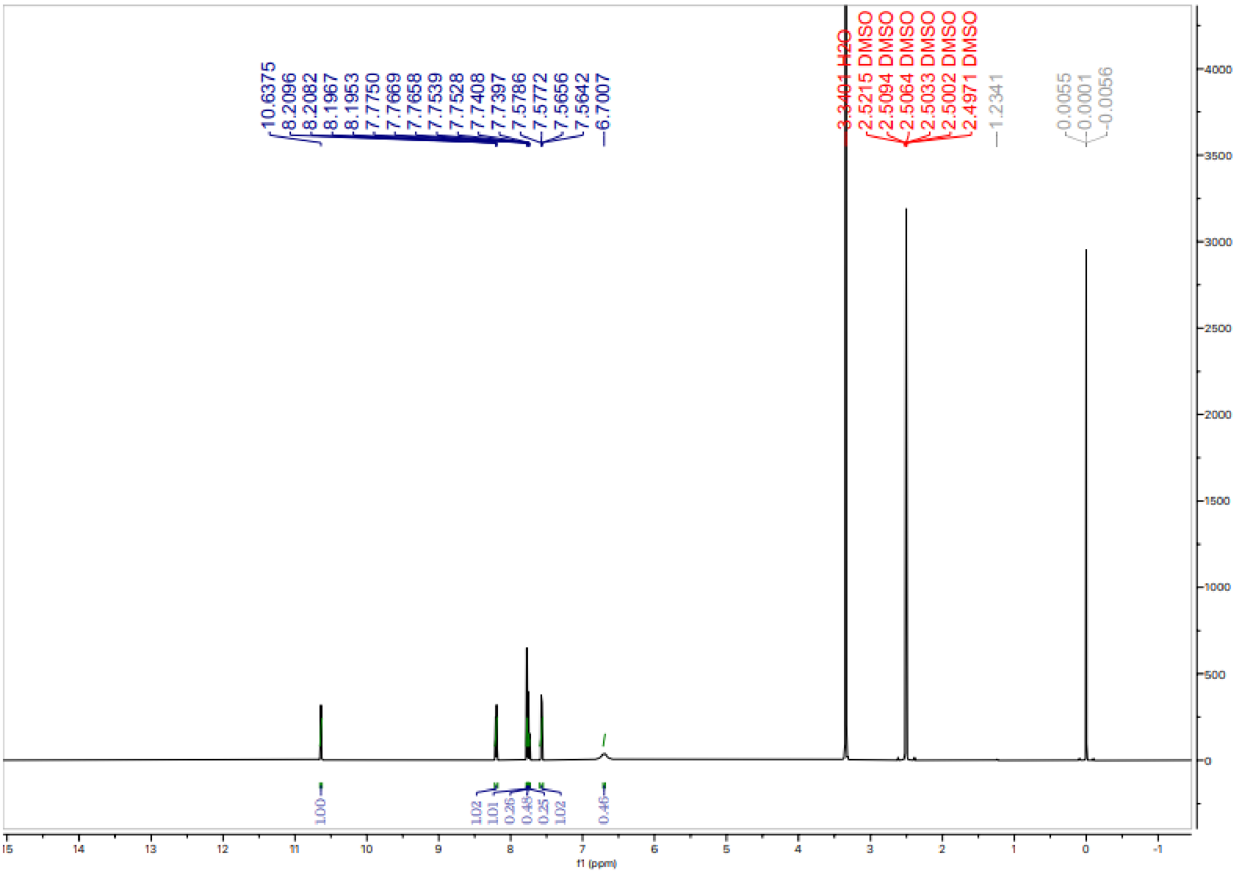
10.6375
8.2096
8.2082
8.1967
8.1953
7.7750
7.7669
7.7658
7.7539
7.7528
7.7408
7.7397
7.5786
7.5772
7.5656
7.5642
6.7007
3.3401 H2O
2.5215 DMSO
2.5094 DMSO
2.5064 DMSO
2.5033 DMSO
2.5002 DMSO
2.4971 DMSO
1.2341
0.0055
0.0001
-0.0056
f1 (ppm)

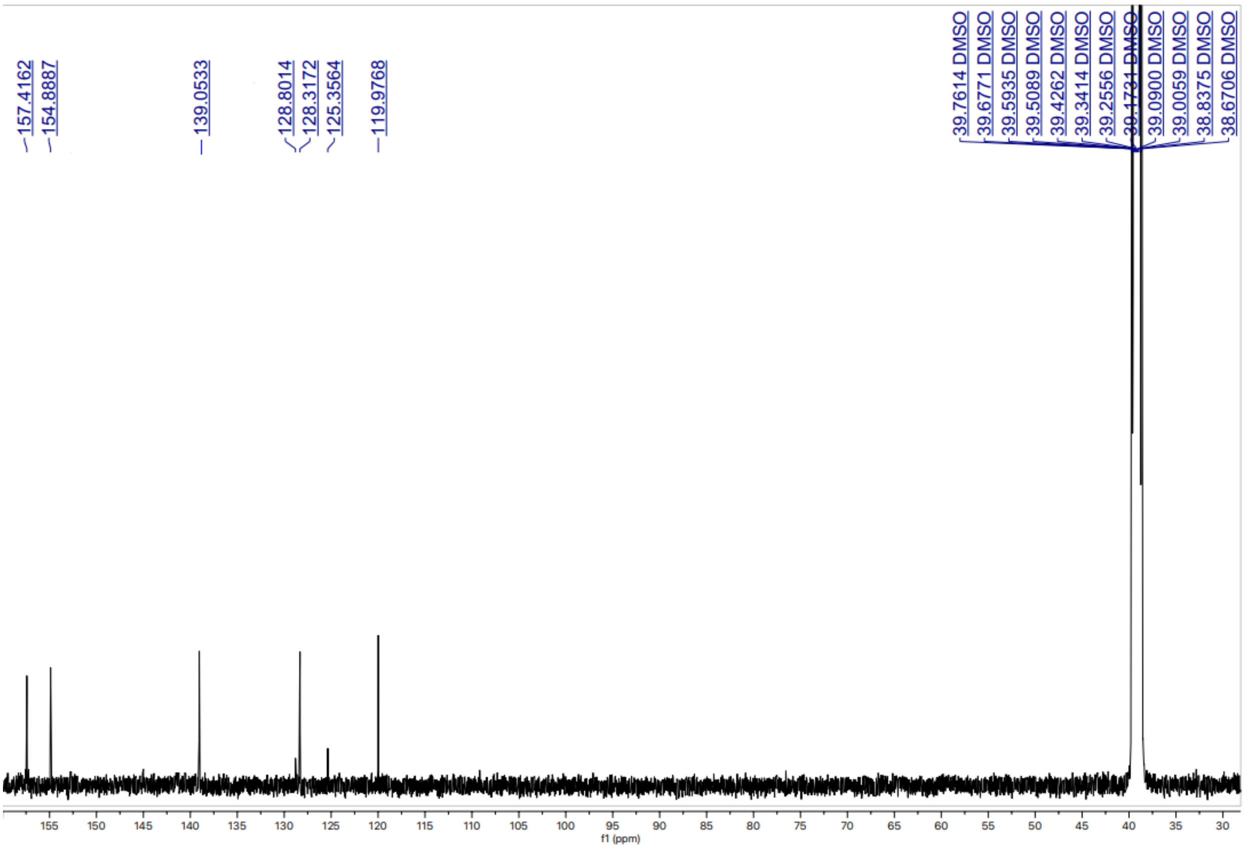
157.4162
154.8887
139.0533
128.8014
128.3172
125.3564
119.9768
39.7614 DMSO
39.6771 DMSO
39.5935 DMSO
39.5089 DMSO
39.4262 DMSO
39.3414 DMSO
39.2556 DMSO
39.1731 DMSO
39.0900 DMSO
39.0059 DMSO
38.8375 DMSO
38.6706 DMSO
f1 (ppm)

RX-10

IR

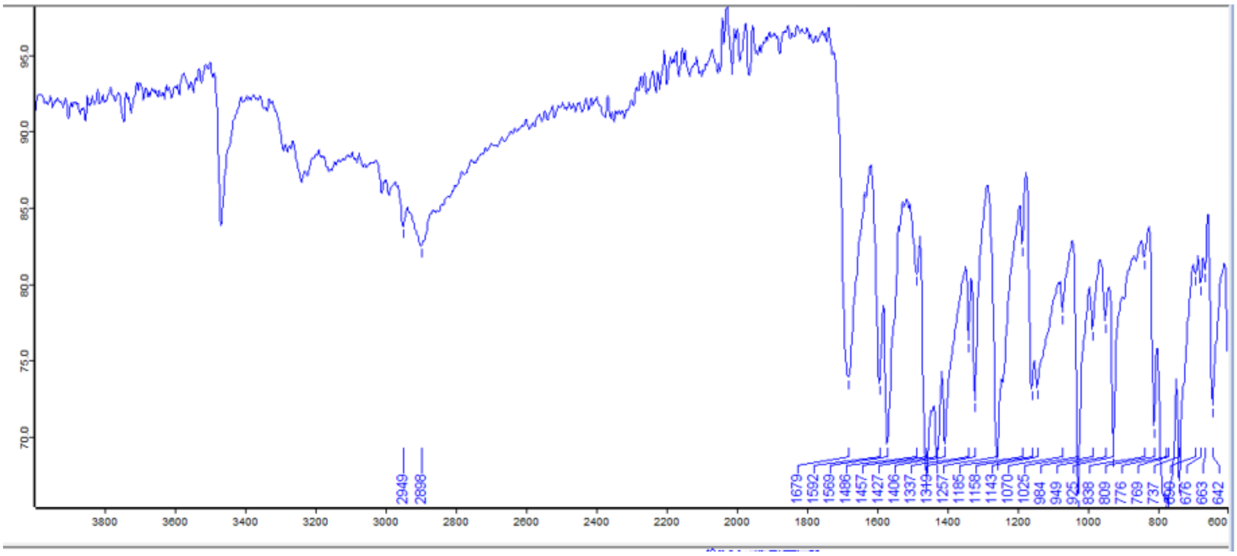
95.0
90.0
85.0
80.0
75.0
70.0
2949
2898
1679
1592
1569
1486
1457
1427
1406
1337
1319
1257
1185
1158
1143
1070
1025
984
949
925
838
809
776
769
737
676
663
642
3800
3600
3400
3200
3000
2800
2600
2400
2200
2000
1800
1600
1400
1200
1000
800
600

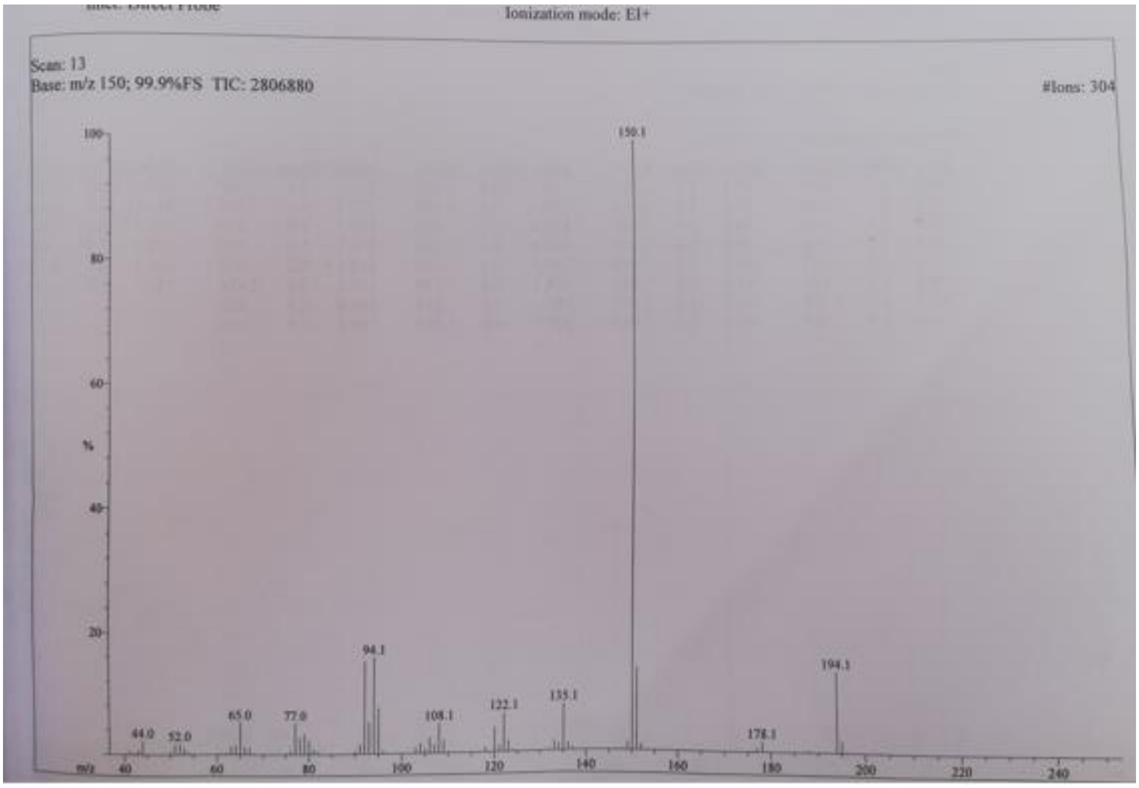
Ionization mode: EI+
Scan: 13
Base: m/z 150; 99.9%FS TIC: 2806880
#Ions: 304
%
150.1
94.1
194.1
135.1
122.1
108.1
77.0
65.0
52.0
44.0
178.1
m/z

NMR

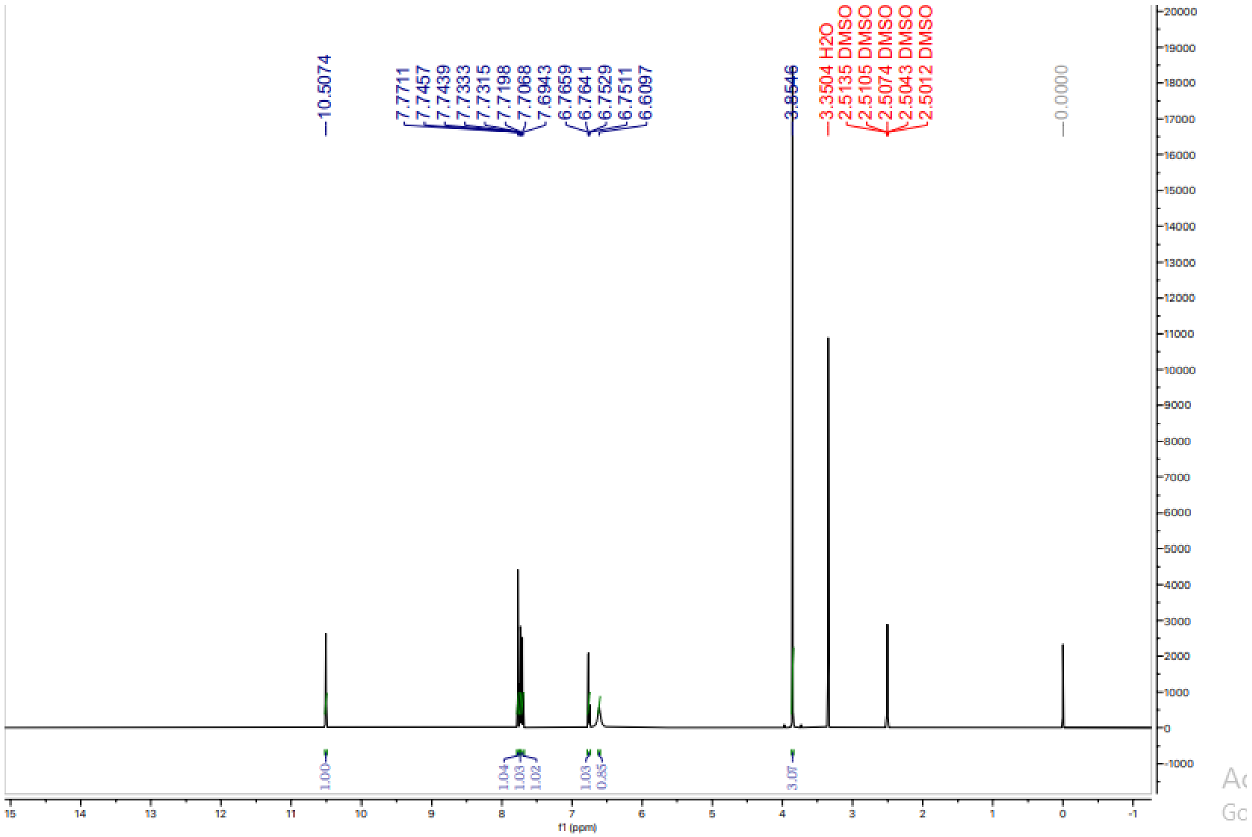

10.5074
7.7711
7.7457
7.7439
7.7333
7.7315
7.7198
7.7068
7.6943
6.7659
6.7641
6.7529
6.7511
6.6097
3.8546
3.3504 H2O
2.5135 DMSO
2.5105 DMSO
2.5074 DMSO
2.5043 DMSO
2.5012 DMSO
0.0000
f1 (ppm)

Rx-11

IR

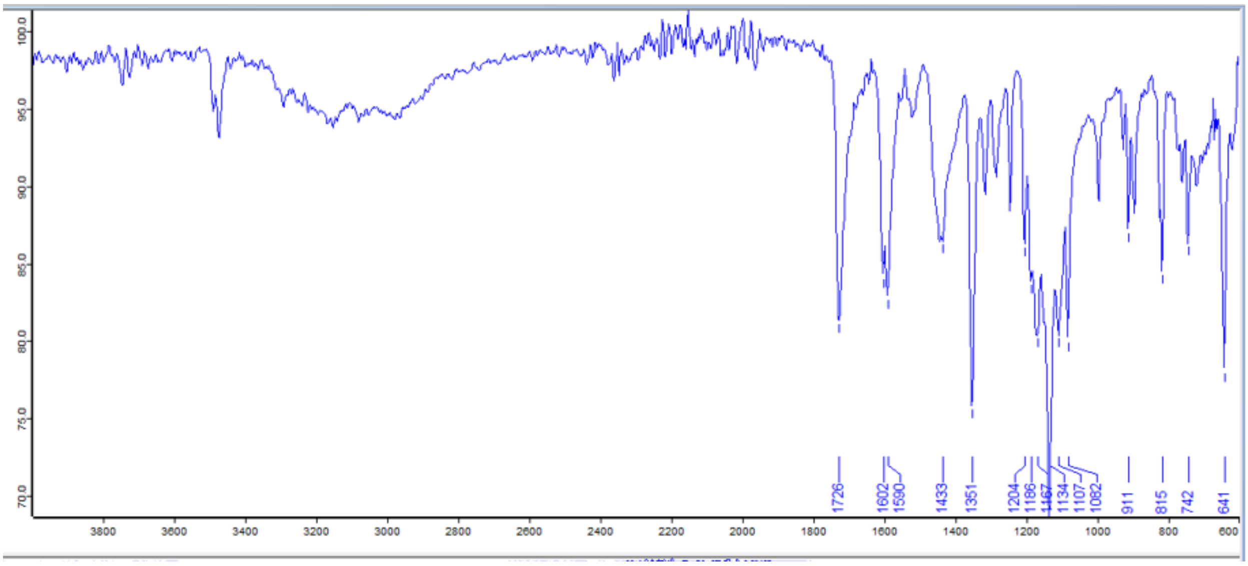

1726
1602
1590
1433
1351
1204
1186
1167
1134
1107
1082
911
815
742
641

Mass

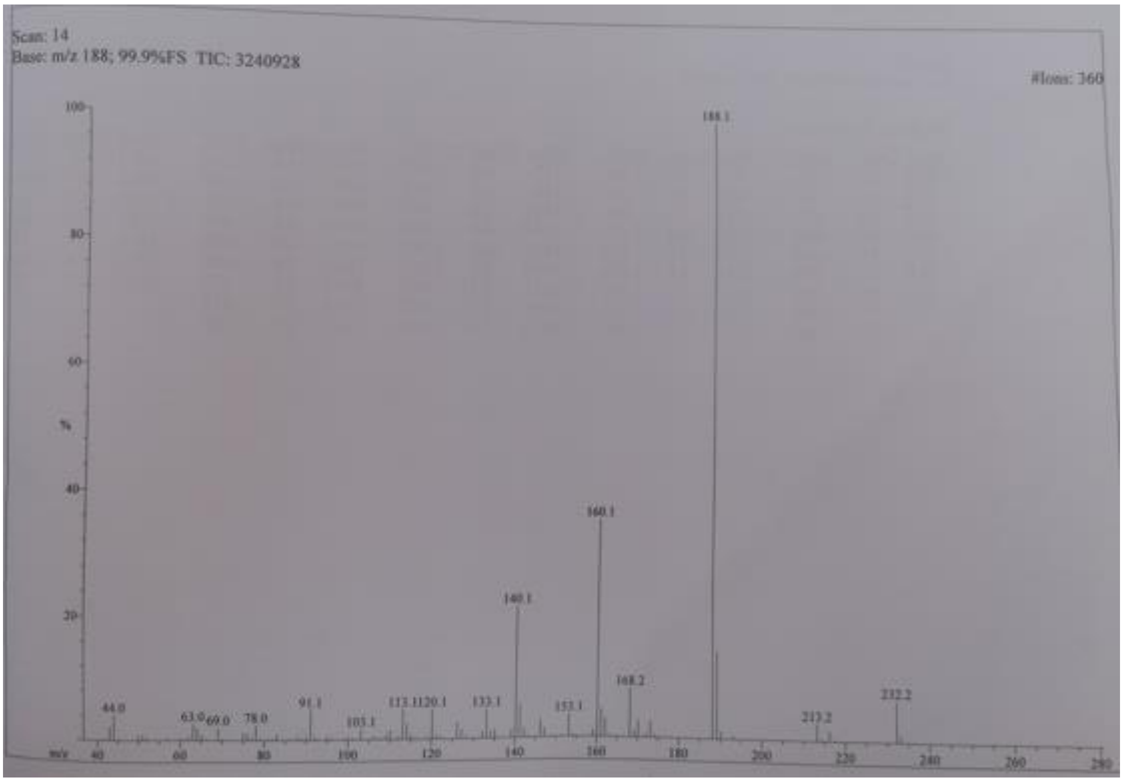
Scan: 14
Base: m/z 188; 99.9%FS TIC: 3240928
#Ions: 360
188.1
160.1
140.1
168.2
212.2
213.2
44.0
63.0
69.0
78.0
91.1
103.1
113.1
120.1
133.1
153.1

NMR

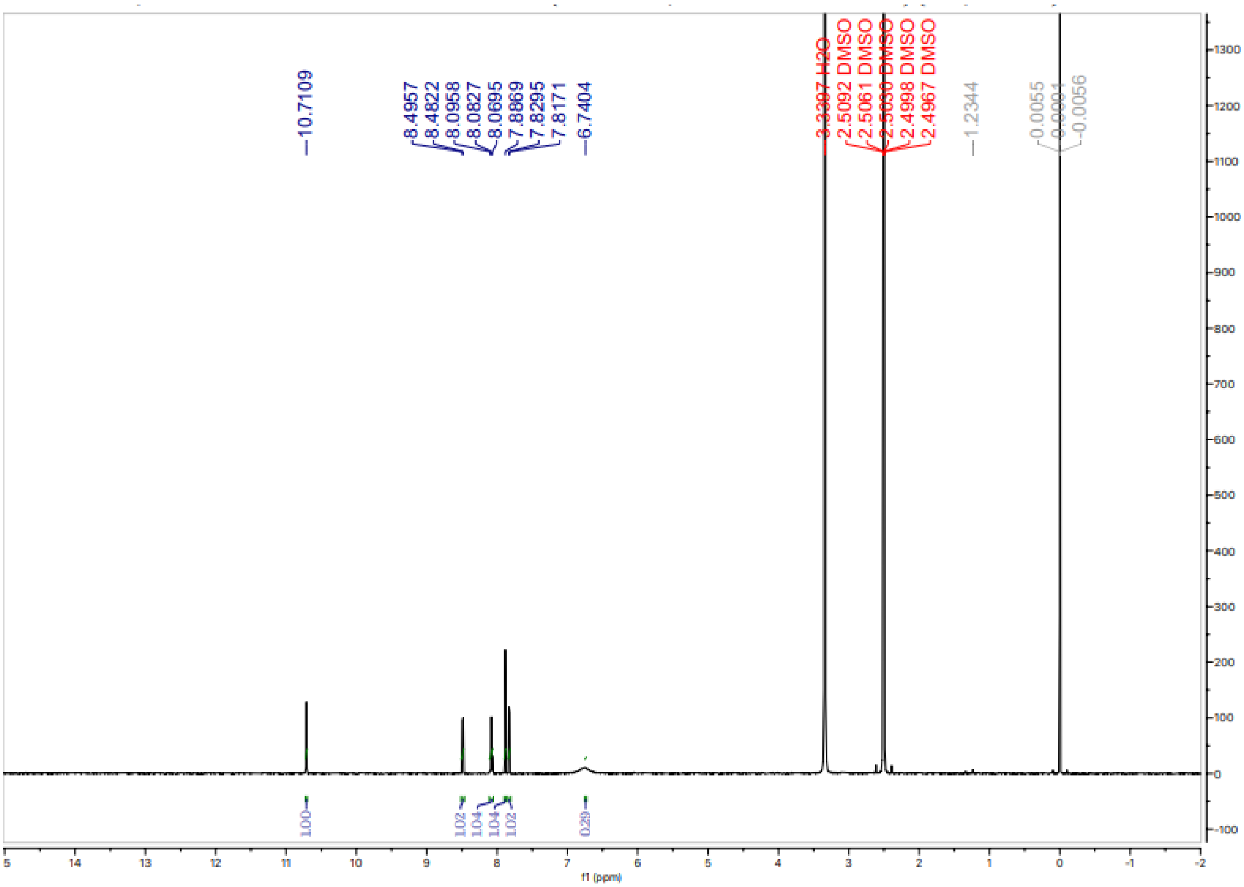
10.7109
8.4957
8.4822
8.0958
8.0827
8.0695
7.8869
7.8295
7.8171
6.7404
2.5092 DMSO
2.5061 DMSO
2.4998 DMSO
2.4967 DMSO
1.2344
0.0055
-0.0056
1.00
1.02
1.04
1.04
1.02
0.29
f1 (ppm)

Rx-12

IR

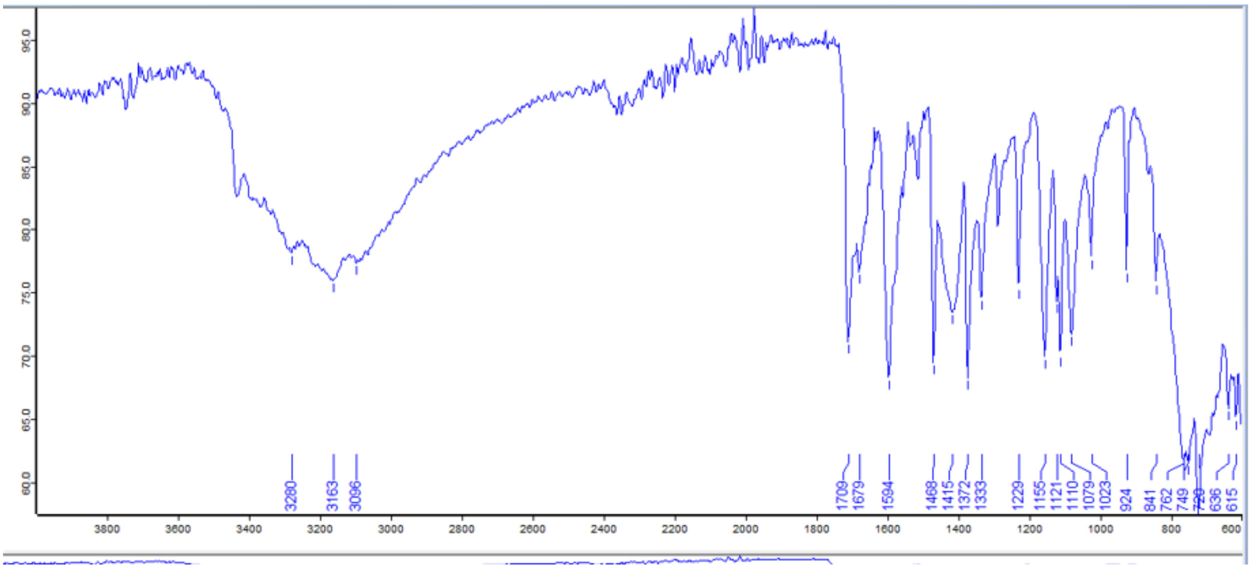
3280
3163
3096
1709
1679
1594
1468
1415
1372
1333
1229
1155
1121
1110
1079
1023
924
841
762
749
636
615

Mass

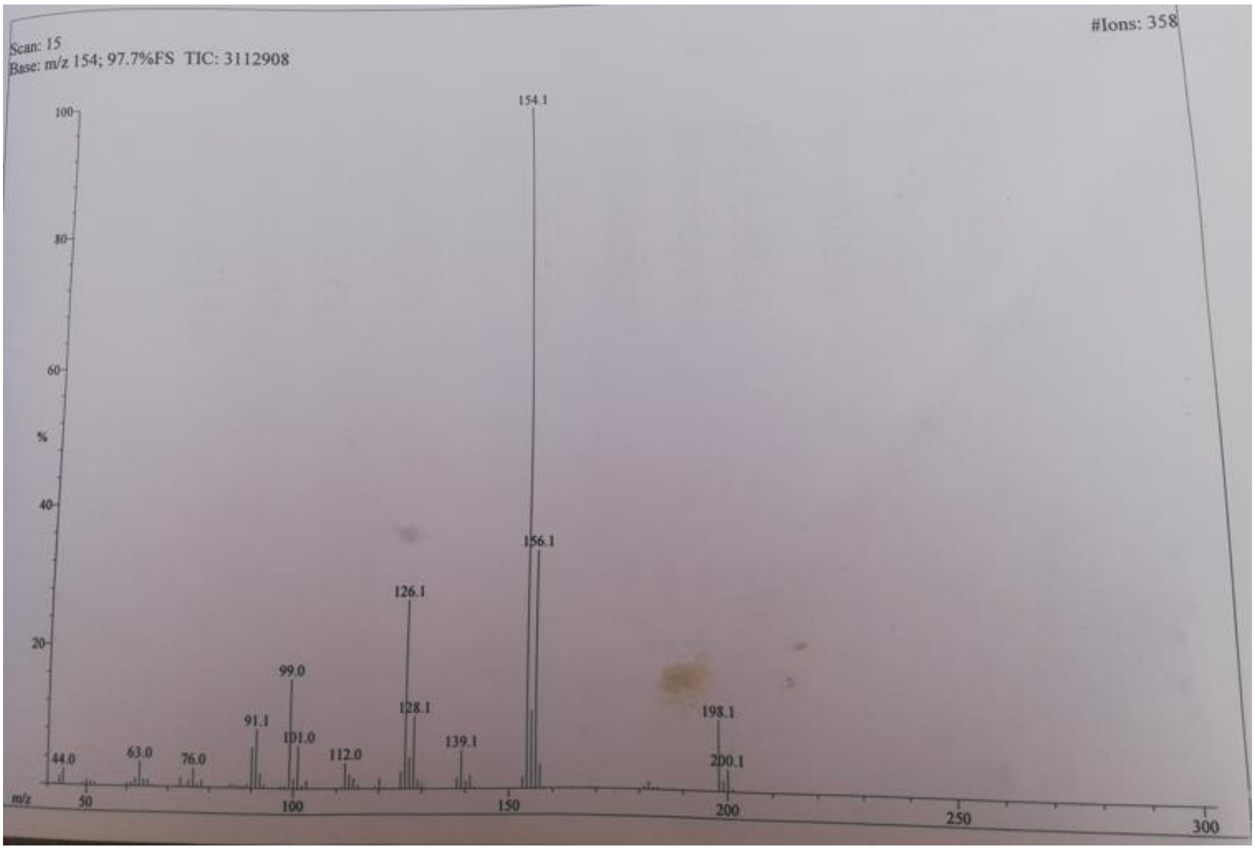
Scan: 15
Base: m/z 154; 97.7%FS TIC: 3112908
#Ions: 358
154.1
156.1
126.1
99.0
128.1
91.1
198.1
101.0
139.1
112.0
200.1
44.0
63.0
76.0

NMR

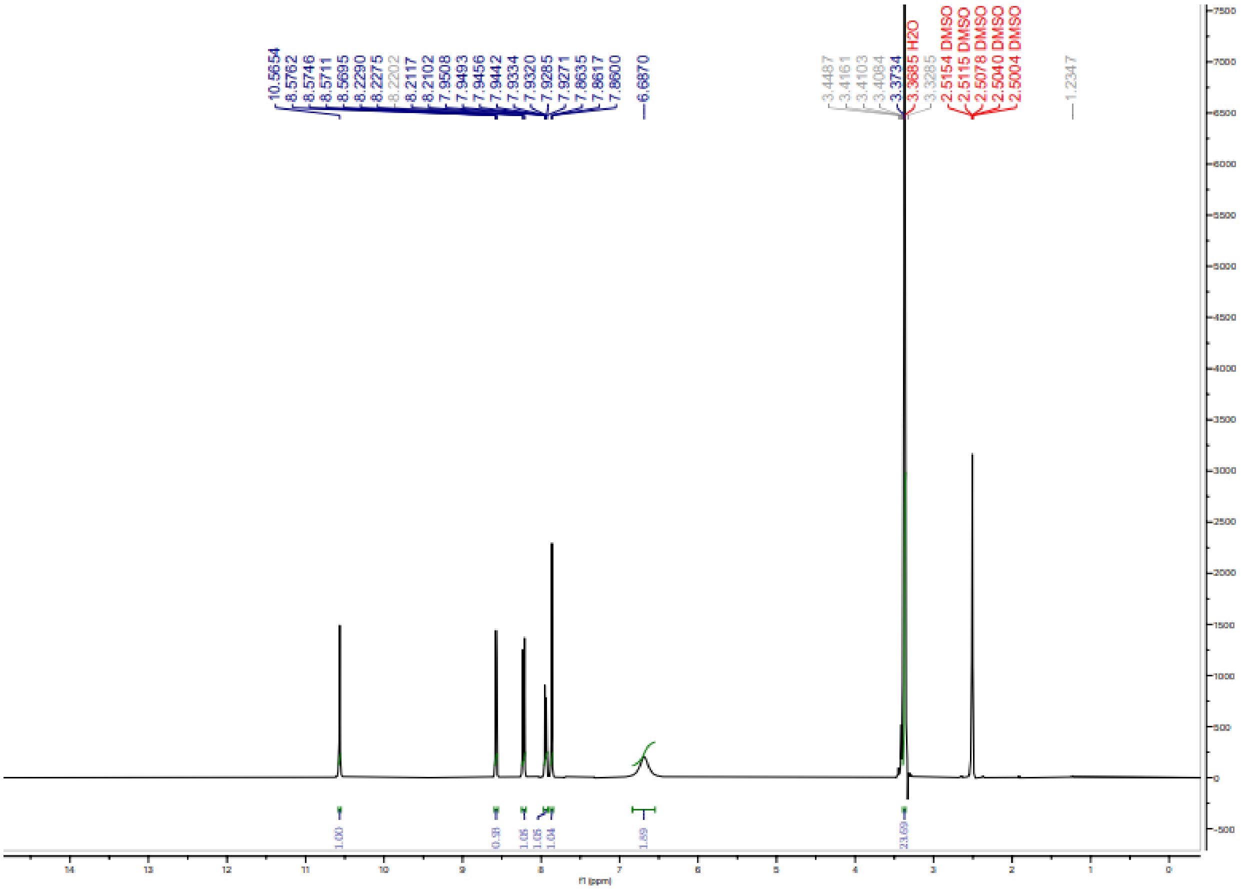
10.5654
8.5762
8.5746
8.5711
8.5695
8.2290
8.2275
8.2202
8.2117
8.2102
7.9508
7.9493
7.9456
7.9442
7.9334
7.9320
7.9285
7.9271
7.8635
7.8617
7.8600
6.6870
3.4487
3.4161
3.4103
3.4084
3.3734
3.3685 H2O
3.3285
2.5154 DMSO
2.5115 DMSO
2.5078 DMSO
2.5040 DMSO
2.5004 DMSO
1.2347
1.00
0.91
1.05
1.05
1.04
1.89
23.69
f1 (ppm)

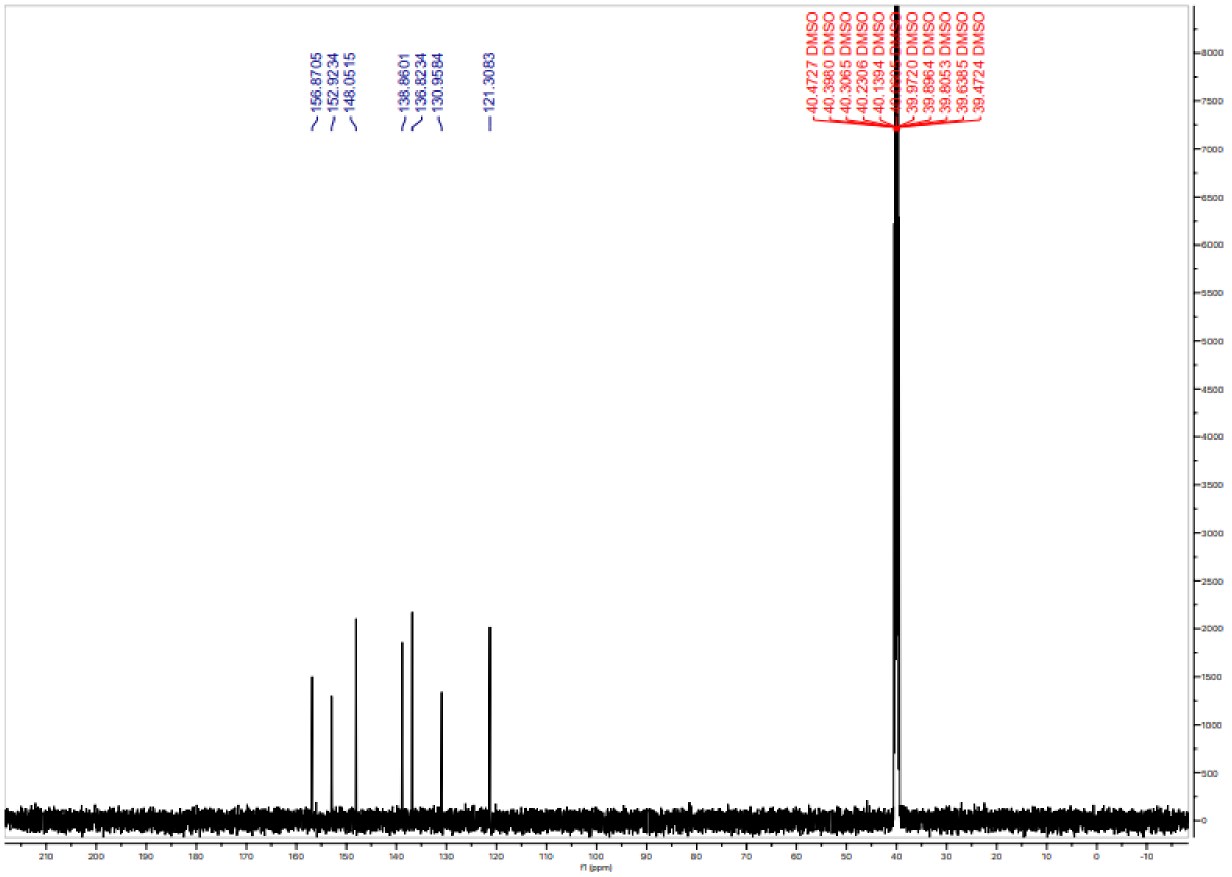
156.8705
152.9234
148.0515
138.8601
136.8234
130.9584
121.3083
40.4727 DMSO
40.3980 DMSO
40.3065 DMSO
40.2306 DMSO
40.1394 DMSO
39.9720 DMSO
39.8964 DMSO
39.8053 DMSO
39.6385 DMSO
39.4724 DMSO
f1 (ppm)
